# Unveiling the Excited‐State Dynamics and Interfacial Interactions in Dye‐Sensitized NaNdF_4_ Nanoparticles for Efficient Photothermal Effect

**DOI:** 10.1002/advs.202503110

**Published:** 2025-05-02

**Authors:** Jiacheng Gong, Wusen Zhou, Zhuo Li, Xingjun Li, Wen Yuan, Xiaobo Gu, Qianqian Niu, Yan Liu, Jin Xu, Renfu Li, Datao Tu, Shan Lu, Xueyuan Chen

**Affiliations:** ^1^ State Key Laboratory of Structural Chemistry and Fujian Key Laboratory of Nanomaterials Fujian Institute of Research on the Structure of Matter, Chinese Academy of Sciences Fuzhou Fujian 350002 China; ^2^ Fujian Science & Technology Innovation Laboratory for Optoelectronic Information of China Fuzhou Fujian 350108 China; ^3^ Fujian College University of Chinese Academy of Sciences Fuzhou Fujian 350002 China; ^4^ College of Chemistry and Materials Science Fujian Normal University Fuzhou Fujian 350007 China; ^5^ Molecular Electronics department Max Planck Institute for Polymer Research Ackermannweg 10 55128 Mainz Germany

**Keywords:** dye sensitization, energy transfer, interfacial interaction, lanthanide nanoparticle, photothermal effect

## Abstract

Near‐infrared (NIR) dyes can overcome the weak absorption of lanthanide nanoparticles (NPs) by antenna sensitization, offering new avenues to develop efficient and versatile lanthanide nanomaterials. However, current research on dye‐sensitized lanthanide NPs for photothermal conversion is still preliminary, and the involved excited‐state dynamics and interfacial interactions remain elusive. Herein, steady‐state/transient absorption spectroscopy and theoretical calculation are used to investigate the coordination and aggregation states of cypate dyes on NaNdF_4_ NPs, revealing the influence of interfacial interactions on resonant energy transfer. Synergetic heat‐generation mechanism of lanthanide cross‐relaxation and dye intermolecular collisions is further proposed. The photothermal conversion efficiency of cypate‐NaNdF_4_ nanocomposites reaches 50.4%, outperforming those of typical photothermal materials with high NIR absorption. Moreover, the intersystem crossing of cypate can be inhibited due to the depopulation of the S_1_ exciton via ET, thereby improving anti‐photobleaching ability. These dye‐sensitized NaNdF_4_ nanocomposites exhibit superior photothermal effect, stability and NIR‐II luminescence, showing great potential in theranostic applications.

## Graphical abstract

A new class of efficient photothermal agents based on dye‐sensitized NaNdF_4_ is developed. The coordination and aggregation states of cypate dyes on lanthanide nanoparticles are identified, revealing the influence of interfacial interactions on resonant energy transfer and synergetic heat generation.

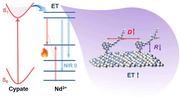

## 1 Introduction

Nd^3+^ highly doped nanoparticles (NPs) exhibit high photothermal conversion efficiency (PCE) owing to their serious cross‐relaxation (CR, ^4^F_3/2_‐^4^I_15/2_ and ^4^I_9/2_‐^4^I_15/2_) and surface quenching effect, being promising candidates for photothermal therapy.^[^ ^1^, ^2^, ^3^, ^4^, ^5^, ^6^, ^7^, ^8^, ^9^, ^10^, ^11^, ^12^, ^13^ ^]^ Typically, NaNdF_4_ was reported as a nano‐heater with PCE up to 85%.^[^ ^12^ ^]^ However, weak absorption of Nd^3+^ ions, which is attributed to lanthanide parity‐forbidden nature, impedes the electron population and actual temperature enhancement even if Nd^3+^ based NPs have noticeable PCE, hampering the materials’ extensive applications.

Near‐infrared (NIR) dyes possess large absorption cross‐section, several orders of magnitude higher than that of lanthanide NPs. A suitable combination of NIR dyes and NPs can amplify superior properties of lanthanide ions. In 2012, Hummelen et al. selected IR‐806 as an antenna to sensitize *β*‐NaYF_4_:Yb^3+^,Er^3+^, leading to a dramatic upconversion luminescence (UCL) enhancement by a factor of ≈3300.^[^ ^14^ ^]^ Since then, the dye‐sensitization strategy emerging as an effective method has been extensively studied in the field of lanthanide luminescent nanomaterials.^[^ ^15^, ^16^, ^17^, ^18^, ^19^, ^20^, ^21^, ^22^, ^23^, ^24^, ^25^, ^26^, ^27^, ^28^ ^]^ However, there have been few reports on dye‐sensitized lanthanide NPs for photothermal conversion. It has been found that NIR dyes can directly convert absorbed photon energy into heat due to intermolecular collisions,^[^ ^29^, ^30^ ^]^ but they usually suffer from low PCE with the value ≈20%.^[^ ^31^, ^32^ ^]^ By transferring absorbed energy from dyes to Nd^3+^ based NPs, the sensitized nonradiative pathways in Nd^3+^ can theoretically increase PCE, and the improvement effect highly depends on the energy transfer (ET) efficiency.

In the framework of Förster resonance ET (FRET) theory, the spatial distance between dye and lanthanide NP (*R*_dye‐NP_) governs the ET efficiency by modulating the extent of dipolar coupling.^[^ ^33^, ^34^, ^35^, ^36^, ^37^ ^]^ Numerous research studied the structural factors that influence *R*_dye‐NP_, aiming to improve the ET efficiency. In 2021, Artizzu et al. used transient absorption (TA) spectroscopy to investigate the relaxation time of dyes with varying conformations on lanthanide‐doped CaF_2_, confirming that efficient ET was attained when the molecules were in close proximity to the NP surface.^[^ ^38^ ^]^ Turshatov et al. compared the binding abilities of various cyanine dyes with lanthanide NPs and found that Cy754 exhibited the superior binding ability, outperforming all other dyes in terms of dye sensitization efficiency.^[^ ^39^ ^]^ More recently, Liu et al. achieved efficient triplet‐state (T_1_) sensitization upconversion by modifying coordination sites on dye conjugated structures.^[^ ^40^ ^]^ In addition to the dye structures, the feeding concentration of dyes concerning the interfacial interactions like coordination and aggregation states of dyes on NPs, can also have a strong impact on *R*_dye‐NP_. Nevertheless, there are few studies about the impact of concentration‐dependent interfacial interactions on *R*_dye‐NP_ and ET efficiency. It is even more obscure how the interactions affect the overall photothermal effect of NIR dye‐lanthanide NPs.

Herein, a kind of photothermal composite materials (cypate‐NaNdF_4_) was developed based on dye sensitization. Cypate was selected as a sensitizer to harvest NIR light and transfer the photon energy to Nd^3+^. Density functional theory (DFT) calculations were used to specify the preferred coordination model of cypate on NaNdF_4_ NPs. The concentration‐dependent evolution of coordination states of cypate on NPs was further unveiled by correlating ET efficiency with *R*_dye‐NP_ based on TA spectroscopy and FRET theory. Steady‐state absorption spectroscopy was adopted to investigate the aggregation behavior of cypate on NPs. The mechanism of synergetic heat generation for cypate‐NaNdF_4_ was proposed. Finally, we explored the potential of the water‐soluble nanocomposites for phototheranostic applications.

## 2 Results and Discussion

**Figure 1:**
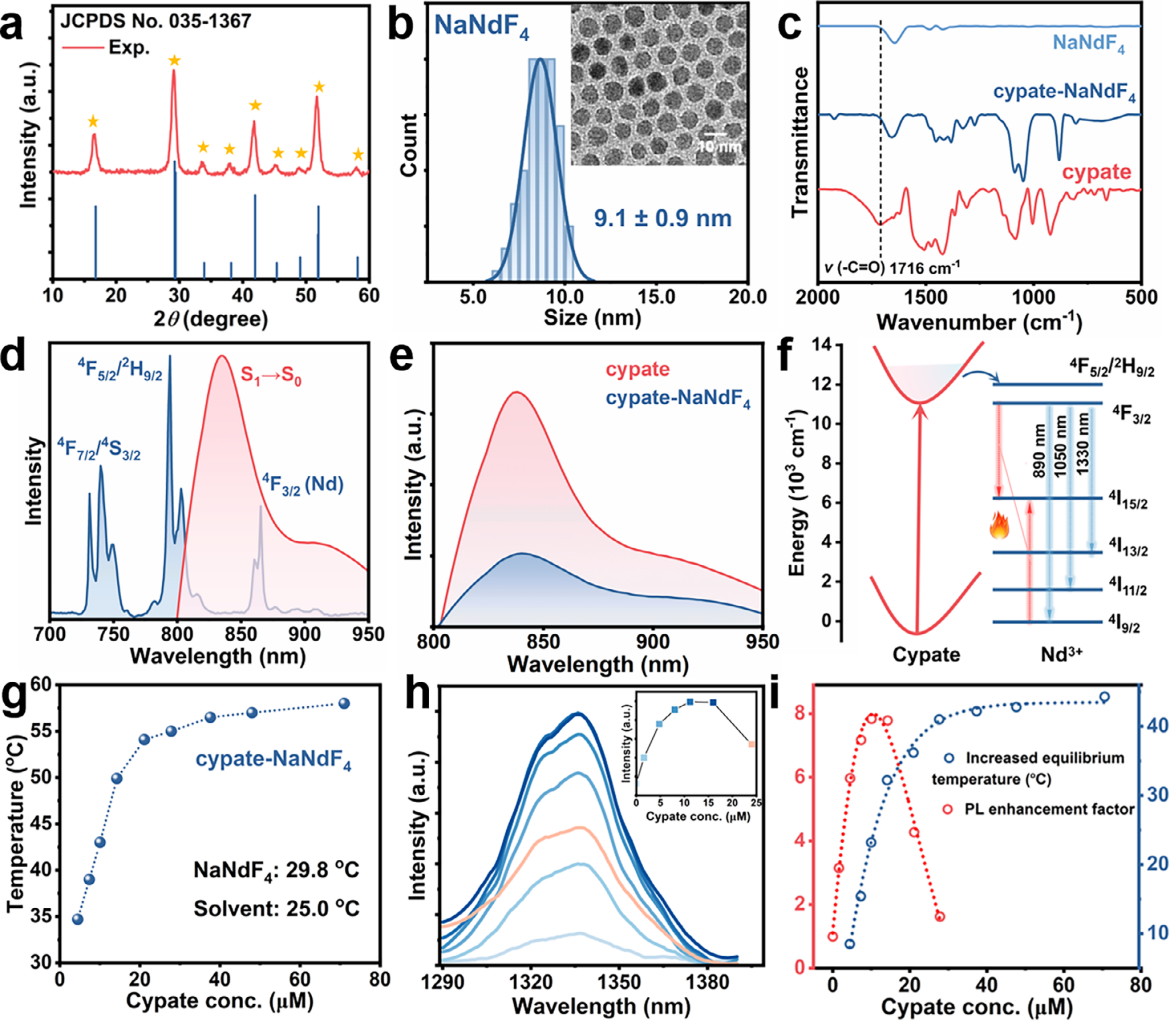
a) PXRD pattern of *β*‐NaNdF_4_. b) TEM image and size distribution histogram of NaNdF_4_ NPs. c) FT‐IR spectra of NaNdF_4_, cypate, and cypate‐NaNdF_4_. d) Emission spectrum of cypate overlapped with the absorption spectrum of NaNdF_4_. e) Emission of cypate before and after binding on NaNdF_4_ under 808‐nm excitation. f) Schematic illustration of ET from cypate to Nd^3+^ for enhancing emission and thermal properties of NaNdF_4_. g) Photothermal equilibrium temperatures of cypate‐NaNdF_4_ composites with 1 mg mL^−1^ of NaNdF_4_ and different concentrations of cypate. h) Sensitized NIR‐II emission of Nd^3+^. Inset: DSL intensity (1330 nm) as a function of the cypate concentration. i) PL enhancement factors and increased equilibrium temperatures of cypate‐NaNdF_4_ with different dye concentrations as compared to NaNdF_4_ alone.

High‐quality NaNdF_4_ NPs were synthesized via the solid‐liquid‐thermal‐decomposition (SLTD) method.^[^ ^41^ ^]^ X‐ray diffraction (XRD) pattern was well assigned to hexagonal phase NaNdF_4_ (PDF#035‐1367) (**Figure** **1**a). Transmission electron microscopy (TEM) revealed that NaNdF_4_ NPs had a uniform spherical morphology with an average size of 9.1 nm (Figure 1b). Cypate, an ICG derivative with high molar extinction coefficient bearing two carboxyl groups, was synthesized and chosen as a NIR‐absorbing antenna (Figures S1‐S4, Supporting Information).^[^ ^42^, ^43^ ^]^ Fourier transform infrared (FT‐IR) spectroscopy confirmed the binding of cypate on the surface of NaNdF_4_ through carboxyl groups (Figure 1c). The exact amount of cypate in the cypate‐NaNdF_4_ composite was determined by steady‐state absorption spectroscopy (Figure S4 and Table S1, Supporting Information). The broad emission of cypate, peaking at 830 nm, presented an overlap with the absorption of the ^4^I_9/2_→^4^F_5/2_ + ^2^H_9/2_ and ^4^I_9/2_→^4^F_3/2_ transitions of Nd^3+^ (Figure 1d).^[^ ^16^ ^]^ The sharply quenched fluorescence of cypate after binding onto NaNdF_4_ implied the occurrence of the ET from cypate to NaNdF_4_ (Figure 1e; Figure S5, Supporting Information). The mechanism behind the sensitization was illustrated in Figure 1f. Upon 808‐nm excitation, singlet excitons (S_1_) formed in excited state, followed by transferring energy to the adjacent ^4^F_5/2_ and ^4^F_3/2_ energy levels of Nd^3+^. The excited electrons quickly relaxed to ^4^I_9/2_, ^4^I_11/2_, and ^4^I_13/2_ via radiative pathways by emitting photons at 890, 1050, and 1330 nm, respectively (Figure S6, Supporting Information).^[^ ^12^, ^44^ ^]^ Transitions of ^4^F_3/2_→^4^I_15/2_ and ^4^I_9/2_→^4^I_15/2_ served as CR pathways, where the energy was subsequently dissipated as heat through nonradiative relaxations.^[^ ^8^ ^]^ Under 808‐nm NIR radiation with a power density of 500 mW cm^−2^, the temperature of NaNdF_4_ dispersion (1 mg mL^−1^) gradually increased and reached a plateau (equilibrium temperature) after ≈3 min (Figure S7, Supporting Information). The equilibrium temperature of NaNdF_4_ dispersion was only raised by ≈5 °C as compared to that of the solvent. In contrast, the equilibrium temperature of cypate‐NaNdF_4_ exhibited a significant increase with the addition of cypate, eventually reaching a high value of 58 °C (Figure 1g). Owing to dye sensitization, the down‐shifting luminescence (DSL) at 1330 nm (from ^4^F_3/2_ to ^4^I_13/2_) was also enhanced despite strong CR effect.^[^ ^45^ ^]^ Specifically, the DSL intensity steadily increased as the cypate concentration increased from 0 to 11.2 µM and then declined due to the fluorescence quenching of cypate at higher concentrations (Figure 1h).^[^ ^27^ ^]^ Both luminescent and thermal properties of lanthanide NPs were improved by increasing the population of electrons in the excited state, which exhibited different cypate concentration‐dependent variations (Figure 1i). To shed light on the dynamic associations among ET, crucial interface interactions, and photothermal performance, we carried out a series of transient and steady‐state spectroscopy analysis on free and bound cypate dyes. Optically inert NaGdF_4_ NPs with a similar particle size to NaNdF_4_ were also synthesized in order to better reveal the effect of coordination and intermolecular interactions in the cypate‐NP composites (Figures S8–S10, Supporting Information).^[^ ^46^, ^47^ ^]^

**Figure 2:**
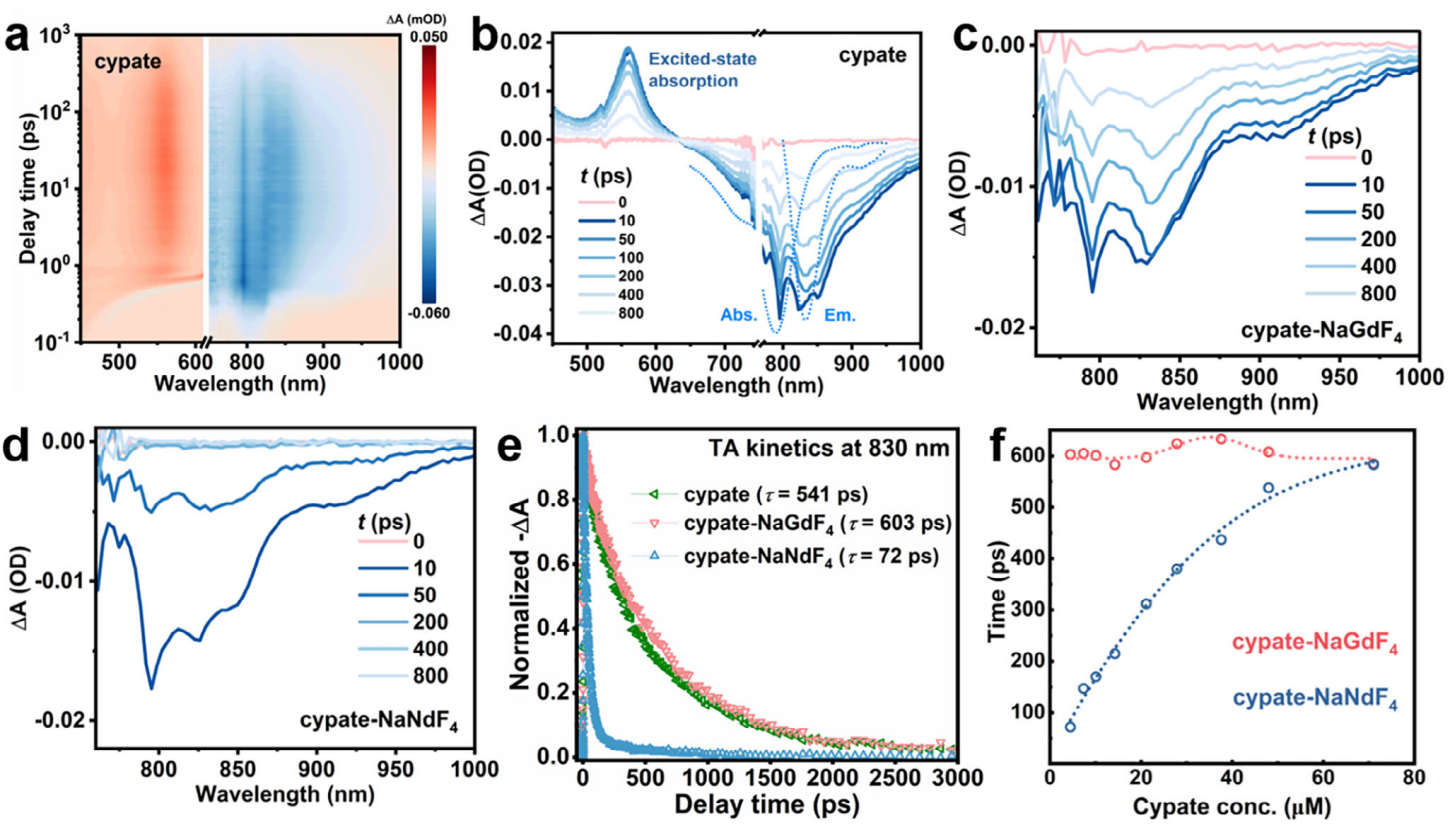
a) fs‐TA map of cypate pumped at 790 nm. Representative time‐resolved spectra of b) cypate, c) cypate‐NaGdF_4_, and d) cypate‐NaNdF_4_. e) TA kinetic traces of free cypate, cypate‐bound NaGdF_4_ and NaNdF_4_ NPs probed at 830 nm. f) Relaxation time of cypate on NaGdF_4_ and NaNdF_4_ NPs as a function of the cypate concentration (NPs conc. 1 mg mL^−1^).

Both ground‐state absorption and singlet‐state relaxation of cypate follow spin‐allowed transition rules, making it possible to harvest photon and sensitize Nd^3+^ efficiently. The excited dyes usually relax to the ground state within nanoseconds (ns). Femtosecond transient absorption (fs‐TA) spectroscopy was employed to study the ultrafast exciton dynamics of cypate.^[^ ^48^ ^]^ Following pulsed excitation, a positive feature of cypate was observed in TA map at 420–630 nm (Figures S11 and S12, Supporting Information), which were attributed to excited state absorption (ESA) (see Section S1.4 Supporting Information),^[^ ^49^, ^50^, ^51^, ^52^ ^]^ while two negative features were also collected and assigned to ground state bleaching (GSB) and stimulated emission (SE) respectively (**Figure** **2**a).^[^ ^27^ ^]^ GSB and SE signals peaking at 780 and 830 nm were in line with singlet exciton formation (S_0_→S_1_) and relaxation (S_1_→S_0_), which can be further confirmed by steady‐state absorption and PL spectrum (Figure 2b). The exciton formation of cypate completed within 1 picosecond (ps), followed by the relaxation of the excited‐state energy for a few ns (Figure 2a,b; Figures S13 and S14, Supporting Information).^[^ ^27^ ^]^ When cypate was bound to optically inert NaGdF_4_ NPs, the relaxation dynamics of cypate showed no obvious change (Figure 2c). In contrast, the SE signal of cypate on NaNdF_4_ NPs was substantially reduced in the same time range. Such an accelerated relaxation indicated the activation of an efficient relaxation channel for S_1_ states of cypate and confirmed the existence of ET from cypate to NaNdF_4_ (Figure 2d).^[^ ^38^ ^]^ For the composites containing 4.5 µM of cypate and 1 mg mL^−1^ of NPs, the relaxation time of cypate was shortened from 603 (on NaGdF_4_) to 72 ps (on NaNdF_4_), indicating of a high ET efficiency of 88% (Figure 2e). The binding of more cypate to NaGdF_4_ NPs caused insignificant changes in the relaxation time of the S_1_ exciton (≈600 ps). While continuous increase of cypate bound to NaNdF_4_ prolonged the relaxation time from 72 to 583 ps, which was closer to the time observed for cypate on NaGdF_4_ (Figure 2f; Table S2, Supporting Information). These results demonstrated that highly concentrated cypate hindered the relaxation path of S_1_ excitons, leaving excited‐state energy trapped in high‐lying states.

**Figure 3:**
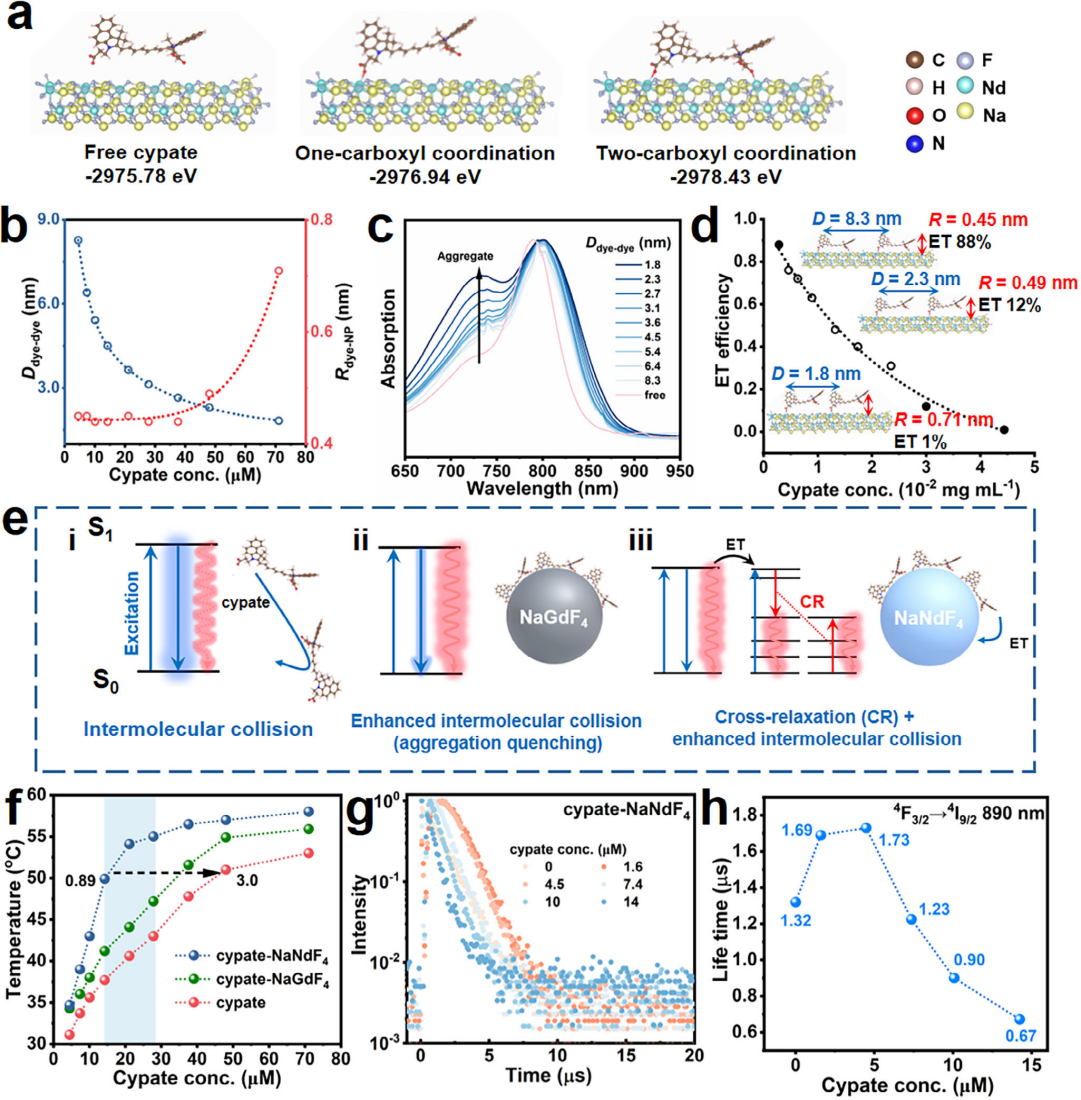
a) The molecular coordination models with the energies of cypate in free state, or bound onto NaNdF_4_ surface by one/two‐carboxyl coordination. b) Cypate concentration‐dependent intermolecular distance (*D*_dye‐dye_) and spatial distance (*R*_dye‐NP_) c) Steady‐state absorption spectroscopy of cypate‐NPs. d) ET efficiency as a function of cypate concentration. The inset depicts the dye in different coordination states. e) Schematics of photothermal conversion mechanisms for cypate, cypate‐NaGdF_4_ and cypate‐NaNdF_4_, respectively: (i) intermolecular collisions of free cypate; (ii) aggregation quenching of cypate on NaGdF_4_; (iii) dye‐sensitized CR of NaNdF_4_ and enhanced intermolecular collisions of cypate. f) Cypate concentration‐dependent photothermal temperature of cypate‐NPs composed of 1 mg mL^−1^ of NaNdF_4_ (or NaGdF_4_) and x µM of cypate. g) PL decay traces of Nd^3+^ at 890 nm with different amount of cypate. h) Cypate concentration‐dependent PL lifetime of ^4^F_3/2_ of Nd^3+^.

To understand the impact of dye concentration and interfacial interactions on ET efficiency, the coordination and aggregation states of cypate on NaNdF_4_ NPs and their influence on *R*_dye‐NP_ were revealed. We first compared the single point energies of molecular models for cypate‐NaNdF_4_ in free or one/two‐carboxyl‐coordination state by performing DFT calculations (**Figure** **3**a).^[^ ^53^ ^]^ The two‐carboxyl‐coordination model showcased greatly reduced energy as compared to the models for free and one‐carboxyl‐coordination states, suggesting that cypate dyes tend to be bound to NPs via two carboxyl groups. FRET model further quantitatively established the correlation between the measured ET efficiency and *R*_dye‐NP_, thereby a series of cypate concentration‐dependent coordination states of cypate on NPs were envisaged. For dye‐sensitized NPs, multiple dye molecules (*n*, the number of donors) are bound to a single NP (as an acceptor). Considering each cypate molecule occupies NaNdF_4_ NP with the number of *n* ^−1^, the FRET efficiency can be expressed as *R*_0_^6^·(*R*_0_^6^+*nR*_dye‐NP_^6^)^−1^.^[^ ^22^, ^54^ ^]^ The emission spectrum and quantum yield for cypate at each concentration were measured to give an accurate estimate of Förster distance, *R*_0_ (Table S3, Supporting Information). *R*_dye‐NP_ at different dye concentrations were obtained accordingly (Figure 3b, see Section S1.3, Supporting Information). Meanwhile, the intermolecular distance between cypate dyes (*D*_dye‐dye_) was estimated based on the exact amount of cypate on NPs (Figure 3b; Table S4, Supporting Information). Cypate dyes arrange in parallel and form aggregates as *D*_dye‐dye_ is small enough,^[^ ^55^, ^56^ ^]^ thus imposing impacts on *R*_dye‐NP_. Steady‐state absorption spectroscopy was used to monitor the formation of cypate aggregates. As illustrated in Figure 3c, cypate displayed a main peak at 795 nm and a shoulder peak at 720 nm, which were assigned to monomers and aggregates, respectively. The signal of aggregates showed a gradual rise with the reduction of *D*_dye‐dye_ and was particularly prominent when *D*_dye‐dye_ was below 2.7 nm. From these results, it can be inferred that cypate molecules in low concentration were tightly bound to NPs by their two carboxyl groups with the *R*_dye‐NP_ of ≈0.45 nm. Cypate maintained the two‐carboxyl‐coordination state as *D*_dye‐dye_ was above 2.7 nm, and the high ET efficiency ranging from 88%‐31% was achieved. As more and more cypate were bound to NPs, the enhanced intermolecular interactions caused one carboxyl group of some cypate molecules detaching from the NPs, extending *R*_dye‐NP_ from 0.45 to 0.49 nm with a decreased ET efficiency to 12%. As *D*_dye‐dye_ was further reduced to 1.8 nm, *R*_dye‐NP_ was extended to 0.71 nm and the ET efficiency dropped to 1% (Figure 3d).

We further explored the photothermal mechanism and optimized the photothermal effect in the cypate‐NaNdF_4_ composites. It was known that cypate dyes can convert absorbed light to heat by intermolecular collisions (Figure 3e‐i). As cypate was bound to NPs, the *D*_dye‐dye_ of cypate on the surface of NPs was shorter than that in the free state, resulting in more frequent molecular collisions and PL quenching (Figure 3e‐ii; Figure S15, Supporting Information).^[^ ^57^ ^]^ Therefore, cypate‐NaGdF_4_ exhibited a slight photothermal enhancement as compared to cypate (Figure 3f). In the case of cypate‐NaNdF_4_, the energy that was not effectively converted to heat by cypate can be extracted by Nd^3+^ through an ultrafast ET process, followed by intense CR process. (Figure 3e‐iii). With the increase of bound cypate, the absorption capacity of cypate‐NaNdF_4_ continued to improve. In the cypate concentration range of 14–28 µM (for 1 mg mL^−1^ of NaNdF_4_), the high absorption and relatively efficient ET (63–40%) of cypate‐NaNdF_4_ facilitated the CR effect of Nd^3+^. The sensitized CR effect together with intermolecular collisions between cypate dyes contributed to the remarkable enhancement of equilibrium temperature (>13.0 °C) as compared to free cypate (Figure 3f). Correspondingly, the PCE increased from 25.8% for cypate, to 35.2% for cypate‐NaGdF_4_, and further to 50.4% for cypate‐NaNdF_4_. (Figure S16 and Table S5, Supporting Information). The PCE value of cypate‐NaNdF_4_ significantly exceeds those of typical photothermal materials like Au, cyanine dye, graphene oxide, etc., which exhibit relatively high absorbance in the NIR region, as detailed in Table S6 (Supporting Information). On the other hand, the dosage of cypate can be effectively reduced. Cypate‐NaNdF_4_ containing 14 µM of cypate displayed an equilibrium temperature of 51.0 °C. This equilibrium temperature can only be reached in free cypate up to 48 µM (Figure 3f).

It should be noted that the photothermal enhancement became less remarkable when the cypate concentration reached 38 µM, even though the ET efficiency was still 31%. Such weakening can be attributed to the energy back transfer (EBT) from NaNdF_4_ to cypate.^[^ ^22^ ^]^ The absorption of cypate presented a partial spectral overlap with the emission of Nd^3+^ at 850–900 nm (Figure S17, Supporting Information), resulting in adverse EBT as excessive cypate dyes were bound to NPs. As shown in Figure 3g,h and Figure S18 (Supporting Information), the PL lifetimes of Nd^3+^ emissions at 890, 1050 and 1330 nm from ^4^F_3/2_ were significantly shortened at relatively high cypate concentrations, confirming the existence of severe EBT. Therefore, it was crucial to meticulously adjust the cypate concentration on NaNdF_4_ so as to ensure effective absorption, high ET efficiency, and minimized EBT process, enabling CR and intermolecular collisions to fully exert their synergetic effect in heat generation.

**Figure 4:**
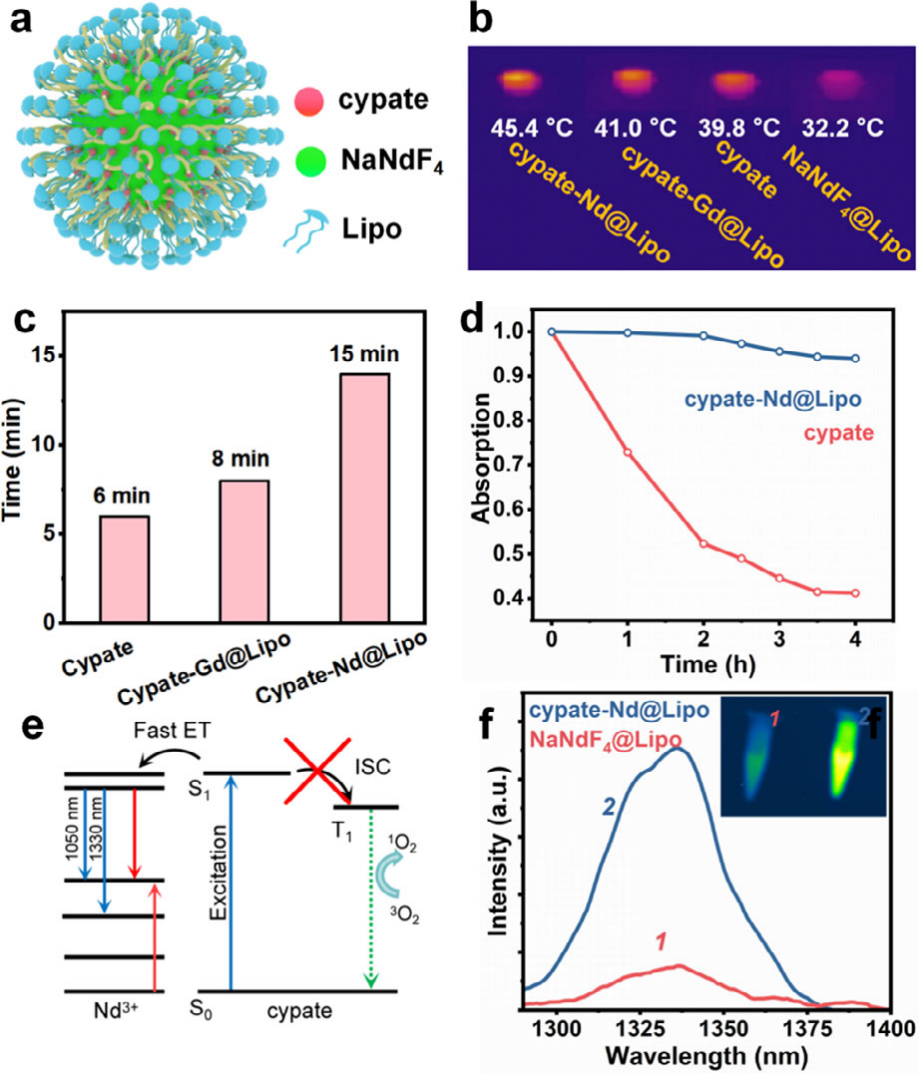
a) Schematic illustration of cypate‐Nd@Lipo. b) Infrared thermal images of NaNdF_4_@Lipo, cypate, cypate‐Gd@Lipo, and cypate‐Nd@Lipo under 808‐nm laser excitation. c) Equilibrium temperatures and duration for cypate, cypate‐Gd@Lipo, and cypate‐Nd@Lipo under 808‐nm laser excitation. d) Changes in the absorption of cypate‐Nd@Lipo and cypate as a function of the exposure time in air. e) Diagram showing the excited‐state energy extracted by Nd^3+^ and reduced ^1^O_2_ generation. f) Cypate‐sensitized NIR‐II luminescence at 1330 nm. Inset depicts the NIR‐II image of NaNdF_4_ and cypate‐Nd@Lipo.

In order to improve the dispersibility of cypate‐NaNdF_4_ in water, amphiphilic phospholipid polymer (Lipo, DSPE‐PEG‐2000) was modified on the surface of cypate‐NaNdF_4_ (cypate‐Nd@Lipo) (**Figure** **4**a; Figure S19, Supporting Information). Dynamic light scattering (DLS) analysis demonstrated that the resulting cypate‐Nd@Lipo showed a narrow hydrodynamic size distribution centered at 20.1 nm and a zeta potential of −15.0 mV (Figures S20 and S21, Supporting Information). Upon 808‐nm radiation with a low power density of 500 mW cm^−2^, cypate‐Nd@Lipo reached an equilibrium temperature of 45.4 °C and remained essentially unchanged for 15 min, while free cypate maintained a temperature of 39.8 °C for only 6 min and dropped dramatically due to photobleaching (Figure 4b,c). We further compared the stability of cypate‐Nd@Lipo and cypate after exposure in air by monitoring their absorption. In a period of 4 h, free cypate degraded by 40%, while cypate‐Nd@Lipo showed little photodegradation (Figure 4d). The improvement of stability can be explained by an energy diagram as shown in Figure 4e. For cypate molecule, following S_1_ exciton formation, part of the excited energy was transferred to the T_1_ state through intersystem crossing (ISC, S_1_→T_1_) pathway.^[^ ^15^, ^21^ ^]^ The T_1_ state then sensitized O_2_ to generate high reactive singlet oxygen (^1^O_2_), causing photocleavage of cypate.^[^ ^58^, ^59^ ^]^ ET (S_1_→Nd^3+^) inhibited the ISC by opening up a depopulating path for the S_1_ exciton, thereby enhancing the dye stability. In addition to the excellent photothermal property, cypate‐Nd@Lipo featured an excellent dye‐sensitized NIR‐II emission (1330 nm, ^4^F_3/2_→^4^I_13/2_) in aqueous media, which makes it an ideal candidate for imaging‐guided photothermal therapy (Figure 4f).^[^ ^60^ ^]^

## 3 Conclusion

In summary, we have developed a new class of photothermal agents based on NIR dye‐sensitized NaNdF_4_ NPs, characterized by both high absorption and remarkable PCE. By means of TA spectroscopy and theoretical calculation, we have identified the coordination and aggregation states of cypate on lanthanide NPs evolved with dye concentration. The analysis has shed light on the critical role that these states play in modulating the spatial distance between dye and lanthanide NP and influencing the ET efficiency. The sensitized CR effect within NaNdF_4_, coupled with increased intermolecular collisions between cypate dyes, has led to a substantial enhancement in photothermal effect. Under 500 mW cm^−2^ 808 nm excitation, cypate‐NaNdF_4_ composites showcased an impressive PCE of 50.4%, exceeding those of the typical photothermal materials with high NIR absorption. Moreover, the ISC of cypate in the composites was inhibited due to the depopulation of the S_1_ exciton via ET, thereby improving the dye's resistance to photobleaching. These findings gain deep insights into the excited‐state dynamics and interfacial interactions in dye‐sensitized lanthanide NPs, which might open up a new avenue for the exploration of efficient photothermal agent toward applications in diverse fields such as phototheranostics.

## 4 Experimental Section

### Chemicals and Materials

Gd(CH_3_COO)_3_·4H_2_O (99.9%), Nd(CH_3_COO)_3_·4H_2_O (99.9%), CH_3_COONa, oleic acid (OA), and 1‐ocatedecence (ODE) were purchased from Sigma–Aldrich (Shanghai, China). Ethanol (EtOH), NaHF_2_, cyclohexane, sodium acetate, acetonitrile and anhydrous ether were purchased from Sinopharm Chemical Reagent Co., China. 1,1,2‐trimethyl‐[1H]benz[e]indole, 3‐bromopropanoic acid, 1,2‐dichlorobenzene, dichloromethane (DCM), acetic anhydride, glutaconaldehyde dianil monohydrochloride, and N,N‐diisopropylethylamine (DIEA) were purchased from Aladdin (Shanghai, China). Deionized (DI) water with resistivity of 18.2 MΩ was used in all experiments.

### Synthesis of *β*‐NaNdF_4_ and *β*‐NaGdF_4_

*β*‐NaNdF_4_ NPs were synthesized via a solid liquid‐thermal decomposition (SLTD) method.^[^ ^41^ ^]^ In a typical process, a mixture of Nd(CH_3_COO)_3_·4H_2_O (1 mmol), CH_3_COONa (4.0 mmol), OA (8 mL), and ODE (12 mL) was heated up to 180 °C under N_2_ flow with vigorous stirring to form a homogenous solution, and then cooled down to room temperature (RT). NaHF_2_ (2.0 mmol) was added. The solution was heated up to 250 °C under N_2_ flow with stirring for 30 min, then increased to 280 °C for 45 min. After the mixture was cooled down to RT, the obtained NPs were precipitated with EtOH, collected via centrifugation, washed with EtOH and cyclohexane for several times, and finally re‐dispersed in cyclohexane for further use. *β*‐NaGdF_4_ NPs were prepared by the same procedure except for Gd(CH_3_COO)_3_·4H_2_O instead of Nd(CH_3_COO)_3_·4H_2_O and 300 °C for 30 min instead of 280 °C for 45 min in crystal growth step.

### Synthesis of Cypate

Cypate was synthesized following the procedure in the literature (Scheme S1, Supporting Information).^[^ ^42^, ^43^ ^]^ First, 1,1,2‐tremethyl‐1H‐benz[e]indole (40.0 g) and 3‐bromopropionic acid (40.0 g) were mixed and reacted in 1,2‐dichlorobenzene (200 mL) with vigorous stirring at 110 °C for 18 h to yield 1,1,2‐tremethyl[1H]‐benz[e]indole‐3‐propanoic acid. In parallel, a mixture of acetic anhydride (1.20 g) and DCM (5 mL) was prepared, and glutaconaldehyde dianilmonohydrochloride (2.85 g) and DIEA (2.60 g) were dissolved in DCM (20 mL) at 0–5 °C. The acetic anhydride/DCM was added dropwise to the glutaconaldehyde/DIEA and stirred for 1.5 h. This mixture was then added to a refluxing acetonitrile/water (95:5) solution containing 1,1,2‐tremethyl[1H]‐benz[e]indole‐3‐propanoic acid (8.2 g) and sodium acetate (3.2 g). The reaction was proceeded at 85 °C for 16 h. After filtration and washing with acetonitrile, 5% HCl, and ethyl ether, ≈6 g (85%) of cypate was obtained.

### Synthesis of cypate‐NPs

The OA‐free NPs (NaNdF_4_ or NaGdF_4_) were obtained by washing the OA‐capped NPs three times with a mixture of cyclohexane, EtOH, DI water, and HCl. Cypate‐NPs was formed by mixing the OA‐free NPs with cypate for 6 h under dark at RT in EtOH, collected by centrifugation and washed twice with EtOH. The loaded amount of cypate in cypate‐NPs was determined by subtracting cypate in the supernatant from cypate added. Since NaNdF₄ and NaGdF₄ had similar crystal structure and particle size (≈9 nm), the difference in their loading amount of cypate was neglected for better comparison (Table S1, Supporting Information).

### Synthesis of Water‐Soluble Nanocomposites

The cypate‐NaNdF_4_ and DSPE‐PEG (10 mg) were added in DCM (4 mL) with vigorous stirring for 8 h. DCM was removed by rotational evaporation. The residue cypate‐NaNdF_4_@DSPE‐PEG (cypate‐Nd@Lipo) was re‐dispersed in 2 mL DI water for further use.

### Photothermal Effect Measurements

250 µL of cypate or cypate‐NPs solution were loaded into 96‐well plate and then continuously irradiated by an 808‐nm laser diode with a power density of 500 mW cm^−2^. As the solution reached an equilibrium temperature, it was naturally cooled. The temperature profile was recorded with a handheld thermal camera (HM‐TPH21Pro‐3AQF, Hangzhou Weiying Software Co., Ltd., HIKMICRO). Photothermal conversion efficiency (PCE, η) was calculated according to the reported method (see Supporting Information 1.2).^[^ ^6^ ^]^

### S_1_ Exciton Relaxation of Cypate

Femtosecond transient absorption (fs‐TA) measurements were conducted under pump wavelength of 790 nm on a Helios (Ultrafast systems) spectrometer using a regeneratively amplified femtosecond Ti:sapphire laser system (Spitfire Pro‐F1KXP, Spectra‐Physics; frequency, 1 kHz; max pulse energy, ≈8 mJ; pulse width, 120 fs) at RT.^[^ ^48^ ^]^ The data were analyzed based on Surface Xplorer (version 4.5.14). Relaxation time of cypate (*τ*_dyes_) was calculated by integrating the kinetic trace^[^ ^15^ ^]^:

(1) $$\tau_{\mathrm{dyes}}=\frac{1}{I_{max}}\int_{0}^{\infty }I\left(t\right)dt$$

where *I*(*t*) denotes the PL intensity as a function of time *t*, and *I_max_* is the maximum PL intensity. Similarly, the relaxation time of cypate on NaGdF₄ or NaNdF_4_ (i.e., *τ*_cypate‐NaGdF4_ or *τ*_cypate‐NaNdF4_) was recorded.

### Single Point Energy

The single point energies were calculated for the cypate‐NaNdF_4_ coordination models, considering both free and one/two‐carboxyl coordinated states. NaNdF_4_ was cleaved along (001) plane. The first‐principle calculations were performed using density functional theory (DFT) within the Vienna ab‐initio simulation package (VASP).^[^ ^61^ ^]^ The exchange‐correlation interaction was processed using a generalized gradient approximation (GGA) in the form of Perdew‐Burke‐Ernzerh of (PBE). A medium precision setting was chosen for the calculation. The kinetic energy cutoff of the plane wave used to extend the Kohn‐Sham electron wave function was set to 400 eV.

### Förster Energy Transfer Model for Calculating R_dye‐NP_

Förster energy transfer (ET) model was available to determine the spatial distance (*R*) between donor and acceptor. The probability (*k*_ET_) of ET was the reciprocal of the sixth power magnitude of *R* ^[^ ^54^ ^]^:

(2) $$k_{ET}=\frac{1}{\tau_{D}}{\left(\frac{R_{0}}{R}\right)}^{6}$$

where τ_*D*_ is the relaxation time of the donor in the absence of acceptor. *R*_0_ is Förster distance. As for dye‐sensitized NPs, multiple dye molecules (*n*, the number of donors) were bound to single NP (an acceptor). This means each cypate molecule occupied NaNdF_4_ NP with the number of *n^−1^*. Therefore, FRTE model can be modified to account for existing several energy donors, and the efficiency of ET can be expressed as^[^ ^54^ ^]^:

(3) $$\mathrm{ET}\;\mathrm{efficiency}\;=\frac{\frac{1}{n}k_{ET}}{\frac{1}{n}k_{ET}+k_{dye}}$$

Recalling *k_ET_*, it can easily rearrange ET efficiency to yield:

(4) $$\mathrm{ET}\;\mathrm{efficiency}\;=\frac{R_{0}^{6}}{R_{0}^{6}+nR_{dye-NP}^{6}}$$

where *R*_0_ =  0.02108[ϕ_dye_ *k* ^2^η^−4^ *J*]^1/6^, η is the refractive index of the medium (taken as 1.4 for DMF in this research); *k* is dipole orientation factor, $\frac{2}{3}$. ϕ_dye_ is the quantum yield of dye molecules in the absence of acceptors. In the research, the quantum yields of cypate on NaGdF_4_ NPs were listed in Table S3 (Supporting Information). *J* (M^−1^ cm^−1^ (nm)^4^) is the spectral overlap integral, as described by,^[^ ^35^ ^]^

(5) $$J=\int F_{dye}\left(\lambda \right)\varepsilon_{Ln}\left(\lambda \right)\lambda^{4}\;d\lambda$$

where *F_dye_* (λ) represents the emission spectrum of cypate normalized to unity, and ε_*Ln*_(λ) is the molar absorptivity (M^−1^ cm^−1^) of NaNdF_4_ NPs. The ET efficiency from cypate to NaNdF_4_ can be determined based on TA by eq. (6),

(6) $$\mathrm{ET}\;\mathrm{efficiency}\;=1-\frac{\tau_{\mathrm{cypate}-\mathrm{NaNdF}4}}{\tau_{\mathrm{cypate}-\mathrm{NaGdF}4}}$$

According to the ET efficiency (Table S2, Supporting Information), a set of cypate concentration‐dependent *R*_dye‐NP_ were successfully calculated (Table S3, Supporting Information).

### Average Intermolecular Distance

The method for calculating average intermolecular distance (*D*_dye‐dye_) can be found in Hummelen et al.’s report (see Section S1.3, Supporting Information).^[^ ^14^ ^]^ Calculated *D*_dye‐dye_ in the study was summarized in Table S4 (Supporting Information).

## Conflict of Interest

The authors declare no conflict of interest.

## Supporting information



Supporting Information

## Data Availability

The data that support the findings of this study are available from the corresponding author upon reasonable request.
