# Supplementary material for: Unveiling the Excited‐State Dynamics and Interfacial Interactions in Dye‐Sensitized NaNdF4 Nanoparticles for Efficient Photothermal Effect
Source: Adv Sci (Weinh). 2025 May 2;12(27):2503110. doi: 10.1002/advs.202503110 (PMC12279181; doi:10.1002/advs.202503110)
Supplement: Supplementary file 1 — Supporting Information [file ADVS-12-2503110-s001.docx]

Supporting Information

Unveiling the excited-state dynamics and interfacial interactions in dye-sensitized NaNdF_4_ nanoparticles for efficient photothermal effect

Jiacheng Gong, Wusen Zhou, Zhuo Li, Xingjun Li, Wen Yuan, Xiaobo Gu, Qianqian Niu, Yan Liu, Jin Xu, Renfu Li, Datao Tu, Shan Lu^*^, and Xueyuan Chen

**1. Experiments**

**1.1 Characterization**

Powder X-ray diffraction (XRD) patterns were collected with X-ray diffractometer (MiniFlex 600, Rigaku) with Cu Kα1 radiation (λ = 0.154 nm), in the 2θ range from 10° to 60°. The size and morphology of *β*-NaGdF_4_ and *β*-NaNdF_4_ NPs were recorded on a TEM (TECNAI G^2^ F20) at an acceleration voltage of 200 kV. The TEM sample was prepared by dripping a NPs cyclohexane solution onto a carbon-coated copper grid and then drying the grid at RT. ^1^H NMR spectrum was recorded on JEOL at 600 MHz. Mass spectrum (MS) was obtained on a Bruker Impact II UHR-TOF instrument. Absorption spectra was recorded on a Perkin-Elmer Lambda 365 UV/Vis/NIR spectrometer. Fourier transform infrared (FTIR) spectra were measured in a Magna 750 FTIR spectrometer from samples in KBr pellets to confirm the success of cypate loading. Zeta potentials and hydrodynamic diameter distributions of various materials dispersed in DI water were determined by means of dynamic light scattering (DLS) measurement (Nano ZS ZEN3600, Malvern). NIR-II PL imaging was collected with a 1100 nm long pass filter and exposure time of 1500 ms under 808-nm excitation (10 W).

Photoluminescence (PL) spectra of lanthanide NPs were recorded by spectrometer (FLS980, Edinburgh) under 808 nm excitation from continuous-wave (cw) semiconductor laser diode. PL spectra of cypate was recorded by same instrument with xenon lamp. The absolute PL quantum yield of cypate was measured by employing a standard barium sulfate coated integrating sphere (150 mm in diameter, Edinburgh) as the sample chamber that was mounted on the FLS980 spectrometer with the entry and output port of the sphere located in 90° geometry from each other in the plane of the spectrometer. The lifetimes of Nd^3+^ were measured with FLS980 equipped with a tunable mid-band Optical Parametric Oscillator (OPO) pulse laser (410-2400 nm, 10 Hz, pulse width 5 ns, Vibrant 355II, OPOTEK).

**1.2 Photothermal conversion efficiency (PCE,** $\eta$**)**

Taking cypate-NaNdF_4_ composed of 21 $\mu$M cypate and 1 mg mL^-1^ NaNdF_4_ for example. PCE value was calculated according to the reported method.^[1]^

$\eta=\frac{hS(T_{max}-T_{surr})-Q_{dis}}{I(1-{10}^{{-A}_{808}})}$ (1)

where *T*_max_ is the equilibrium temperature (54.1 ^o^C), *T*_surr_ is the ambient temperature (40.6 ^o^C), *I* (225 mW) is the incident laser power, and *A*_808_ is the absorbance of solution at 808 nm, 1.26; $Q_{dis}$ associated with the light absorbance of the solvent, which is measured using solvent. $h$ is the heat transfer coefficient, $S$ is the surface area of the container. *hS* is derived on the basis of the following equation:

$hS=-\frac{mC}{\tau_{s}}$ (2)

*m* is the solution mass and equal to 0.24 g in the current study, *C* is the heat capacity of the solution (2.14 J/g for DMF), and $\tau_{s}$ is the associated time constant.

$t=-\tau_{s}ln(\theta)$ (3)

$\tau_{s}$ can be fitted with the linear time from cooling profile versus the negative natural logarithm of the system driving force temperature (Figure S16), which is a dimensionless parameter($\theta$), known as:

$\theta=\frac{T-T_{surr}}{T_{max}-T_{surr}}$ (4)

**1.3 Average intermolecular distance**

Average intermolecular distances were calculated following the method in the literature.^[2]^

**1.3.1 Number of NPs**

Size (*d*) of NPs is found to be 9 nm (from TEM images)

Radius of single NP (*r*) $=\frac{d}{2}=\frac{9}{2} \mathrm{nm}=4.5 \mathrm{nm}$

Volume of single NP (*V*)$=\frac{4}{3}\pi r^{3}=\frac{4}{3}\times\pi\times{4.5}^{3}\mathrm{nm}^{3}=381.51 \mathrm{nm}^{3}$

Weight of single NP (*m*) $=V\rho=381.51\times4.23\times{10}^{-21} g=1.61\times{10}^{-18} g$

We measured the weight of the ligand-free NPs and the ligand weight was excluded in our calculation ($m^{'}$)*.* $m^{'}=$1 mg;

$$n_{\mathrm{NP}}=\frac{m^{'}}{m} =6.20\times{10}^{17}$$

**1.3.2 Number of cypate attached to the surface of NP (taking *C*_cypate_ = 0.00282 mg mL^-1^ for example)**

The weight of cypate was calculated as the loaded content of cypate was obtained, $m_{\mathrm{cypate}}=C_{\mathrm{cypate}}\times1 mL=$0.00282 mg, in the context of 1 mL solution.

Number of cypate $=\frac{m_{\mathrm{cypate}}}{MW}\times N_{A}=\frac{0.00282}{625}\times6.02\times{10}^{21}=2.72\times{10}^{18}$

$$\frac{Number of cypate}{Number of \mathrm{NPs}}=\frac{2.72\times{10}^{18}}{6.20\times{10}^{17}}\approx4$$

**1.3.3 Available area for cypate loading (taking *C*_cypate_ = 0.00282 mg mL^-1^ for example)**

Surface area of single NP (*A*) $=4\pi r^{2}=4\times\pi\times{4.5}^{2}\mathrm{nm}^{2}=254.34 \mathrm{nm}^{2}$

Available area $=\frac{A}{4} =58.02 \mathrm{nm}^{2}$

Average molecular separation (*D*) can be obtained by applying the estimating method: $D=2\sqrt{\frac{A}{\pi}}=2\sqrt{\frac{58.02}{\pi}}=8.27 nm$

**1.4 The origin of ESA in cypate**

To clarify the origin of ESA signal of cypate at around 450 nm (Figure S11), we carried out ultrafast exciton dynamics studies in the range from 420 to 600 nm based on singular value decomposition (SVD) analysis. As shown in Figure S12, two components were extracted based on SVD. The second principal component weighs less and is significantly smaller than the first principal component, suggesting that the pronounced signal ranging from 420-600 nm is mainly contributed by a single component (ESA of S_1_ excitons).

**2. Results and Discussion**

**Table S1**. Loading efficiencies of cypate on NPs at different initial dye concentrations.

| Cypate  (mg mL^-1^) | Cypate  ($\mu$M) | NaGdF_4_  or NaGdF_4_  (mg mL^-1^) | Loading efficiency | Cypate loaded  ($\mu$M) |
| --- | --- | --- | --- | --- |
| 0.003 | 4.8 | 1 | 0.94 | 4.5 |
| 0.005 | 8.0 | 1 | 0.92 | 7.4 |
| 0.007 | 11 | 1 | 0.90 | 10 |
| 0.010 | 16 | 1 | 0.89 | 14 |
| 0.015 | 24 | 1 | 0.88 | 21 |
| 0.020 | 32 | 1 | 0.87 | 28 |
| 0.030 | 48 | 1 | 0.78 | 38 |
| 0.040 | 64 | 1 | 0.75 | 48 |
| 0.060 | 96 | 1 | 0.74 | 71 |

**Table S2.** Kinetic date acquired from TA and calculated ET efficiency.

| Cypate loaded ($\mu$M) | $\tau$_cypate_ (ps) ^1^ | *k*_cypate_ (ps^-1^) ^2^ | $\tau$_cypate-NaGdF4_ (ps) ^3^ | *k*_cypate-NaGdF4_ (ps^-1^) ^5^ | $\tau$_cypate-NaNdF4_ (ps) ^4^ | *k*_cypate-NaNdF4_ (ps^-1^) ^6^ | *k*_ET_  (ps^-1^) ^7^ | ET efficiency ^8^ |
| --- | --- | --- | --- | --- | --- | --- | --- | --- |
| 4.5 | 541 | 0.00185 | 603 | 0.00166 | 72 | 0.0139 | 0.012 | 0.88 |
| 7.4 | 411 | 0.00243 | 605 | 0.00165 | 147 | 0.0068 | 0.005 | 0.76 |
| 10 | 307 | 0.00326 | 601 | 0.00166 | 170 | 0.0059 | 0.004 | 0.72 |
| 14 | 451 | 0.00222 | 583 | 0.00172 | 215 | 0.0047 | 0.003 | 0.63 |
| 21 | 463 | 0.00216 | 597 | 0.00168 | 312 | 0.0032 | 0.002 | 0.48 |
| 28 | 465 | 0.00215 | 624 | 0.00160 | 380 | 0.0026 | 0.001 | 0.40 |
| 38 | 486 | 0.00206 | 633 | 0.00158 | 437 | 0.0023 | 0.0007 | 0.31 |
| 48 | 526 | 0.00190 | 608 | 0.00164 | 538 | 0.0019 | 0.0002 | 0.12 |
| 71 | 515 | 0.00194 | 585 | 0.00171 | 583 | 0.0017 | 0.00002 | 0.01 |

^1^ $\tau$_cypate_ represents the relaxation time of S_1_ excitons of free cypate.

^2^ $k$_cypate_ represents the relaxation rate of S_1_ excitons of free cypate, which is calculated by $\frac{1}{\tau_{\mathrm{cypate}}}$.

^3,4^ $\tau_{cypate-NaGdF4}$and $\tau_{cypate-NaNdF4}$represent the relaxation times of S_1_ excitons of cypate bound on NaGdF_4_ and NaNdF_4_, respectively.

^5,6^ $k_{cypate-NaGdF4}$ and $k_{cypate-NaNdF4}$ represent the relaxation rates of S_1_ excitons of cypate bound on NaGdF_4_ and NaNdF_4_, respectively, calculated by $\frac{1}{\tau_{cypate-NaGdF4}}$ and $\frac{1}{\tau_{cypate-NaNdF4}}$.

^7^ The energy transfer rate, *k*_ET_ can be estimated by: *k*_ET_ = $\frac{1}{\tau_{cypate-NaGdF4}-\tau_{cypate-NaNdF4}}$.

^8^ The ET efficiency can be estimated by: *E* = $1-\frac{\tau_{cypate-NaNdF4}}{\tau_{cypate-NaGdF4}}$ .

**Table S3.** List of calculated *R*_dye-NP_ based on FRET theory.

| Cypate loaded ($\mu$M) | Number ratio *n* (cypate: NP) | Spectral overlap integral (*J*) | Quantum yield | *R*_0_ (nm) | ET efficiency | *R*_dye-NP_ (nm) |
| --- | --- | --- | --- | --- | --- | --- |
| 4.5 | 4.4 | 303186000000 | 0.06 | 0.80 | 0.88 | 0.45 |
| 7.4 | 7.2 | 276661000000 | 0.05 | 0.76 | 0.76 | 0.45 |
| 10 | 9.8 | 255427000000 | 0.05 | 0.75 | 0.72 | 0.44 |
| 14 | 13.9 | 240711000000 | 0.05 | 0.74 | 0.63 | 0.44 |
| 21 | 20.5 | 220460000000 | 0.05 | 0.73 | 0.48 | 0.45 |
| 28 | 27.1 | 228160000000 | 0.04 | 0.71 | 0.40 | 0.44 |
| 38 | 36.5 | 217043000000 | 0.04 | 0.70 | 0.31 | 0.44 |
| 48 | 46.6 | 216148000000 | 0.03 | 0.67 | 0.12 | 0.49 |
| 71 | 69.0 | 217256000000 | 0.03 | 0.67 | 0.01 | 0.71 |

**Table S4**. List of intermolecular distances (*D*_dye-dye_) in different cypate/NP ratios.

| Cypate loaded ($\mu$M) | Surface area of single NP (nm^2^) | Number of NPs | Number of cypate | Number ratio *n* (cypate: NP) | Area for each cypate (nm^2^) | *D*_dye-dye_ (nm) |
| --- | --- | --- | --- | --- | --- | --- |
| 4.5 | 254.3 | 6.2$\times{10}^{17}$ | 2.7$\times{10}^{18}$ | 4.4 | 58.0 | 8.3 |
| 7.4 | 254.3 | 6.2$\times{10}^{17}$ | 4.4$\times{10}^{18}$ | 7.2 | 35.6 | 6.4 |
| 10 | 254.3 | 6.2$\times{10}^{17}$ | 6.1$\times{10}^{18}$ | 9.8 | 26.0 | 5.4 |
| 14 | 254.3 | 6.2$\times{10}^{17}$ | 8.6$\times{10}^{18}$ | 13.9 | 18.3 | 4.5 |
| 21 | 254.3 | 6.2$\times{10}^{17}$ | 1.3$\times{10}^{19}$ | 20.5 | 12.4 | 3.6 |
| 28 | 254.3 | 6.2$\times{10}^{17}$ | 1.7$\times{10}^{19}$ | 27.1 | 9.4 | 3.1 |
| 38 | 254.3 | 6.2$\times{10}^{17}$ | 2.3$\times{10}^{19}$ | 36.5 | 7.0 | 2.7 |
| 48 | 254.3 | 6.2$\times{10}^{17}$ | 2.9$\times{10}^{19}$ | 46.6 | 5.5 | 2.3 |
| 71 | 254.3 | 6.2$\times{10}^{17}$ | 4.3$\times{10}^{19}$ | 69.0 | 3.7 | 1.8 |

**Table S5**. PCEs and equilibrium temperatures.

| Sample | Ambient temperature (^o^C) | Equilibrium temperature (^o^C) | Time constant (s) | Absorbance | *hs* (mW ^o^C^-1^) | PCE |
| --- | --- | --- | --- | --- | --- | --- |
| Cypate | 23.0 | 40.6 | 152 | 1.24 | 3.38 | 25.8% |
| Cypate-NaGdF_4_ | 23.0 | 44.1 | 136 | 1.26 | 3.78 | 35.2% |
| Cypate-NaNdF_4_ | 23.0 | 54.1 | 140 | 1.44 | 3.67 | 50.4% |

**Table S6**. Summary of representative photothermal materials showing relatively high absorbance in the NIR region.

| Material | | Laser wavelength (nm) | Solvent | PCE (%) | Ref. |
| --- | --- | --- | --- | --- | --- |
| cypate-NaNdF_4_ | 808 | | DMF | 50.4 | This work |
| cypate-Nd@Lipo | 808 | | DI water | 45.3 | This work |
| cypate | 785 | | DMSO | 25.6 | ^[3]^ |
| Lipo-cypate | 785 | | DI water | 28.4 | ^[3]^ |
| ICG | 808 | | PBS | 15.1 | ^[4]^ |
| Au stars | 808 | | DI water | 28.0 | ^[5]^ |
| Au vesicles | 808 | | DI water | 37.0 | ^[6]^ |
| Au hexapods | 808 | | DI water | 29.6 | ^[7]^ |
| Au nanorods | 800 | | DI water | 21.0 | ^[8]^ |
| Au nanoshells | 800 | | DI water | 13.0 | ^[8]^ |
| Cu Nanowires | 808 | | DI water | 12.5 | ^[9]^ |
| Cu_2-x_Se | 800 | | DI water | 22.0 | ^[8]^ |
| Ag_2_S | 785 | | DI water | 35.0 | ^[10]^ |
| Bi_2_S_3_ nanorods | 808 | | DI water | 28.1 | ^[11]^ |
| Nb_2_C nanosheets | 808 | | DI water | 36.4 | ^[12]^ |
| Ta_4_C_3_ nanosheets | 808 | | DI water | 44.7 | ^[13]^ |
| Ti_3_C_2_ nanosheets | 808 | | DI water | 30.6 | ^[14]^ |
| TiN | 808 | | DI water | 48.0 | ^[15]^ |
| Prussian blue | 808 | | DI water | 41.4 | ^[16]^ |
| black phosphorus | 808 | | PBS | 38.8 | ^[17]^ |
| graphene oxide sheets | 808 | | DI water | 41.0 | ^[18]^ |
| carbon nanotubes | 808 | | DI water | 39.4 | ^[19]^ |
| BSA–PANI | 808 | | PBS | 37.0 | ^[20]^ |


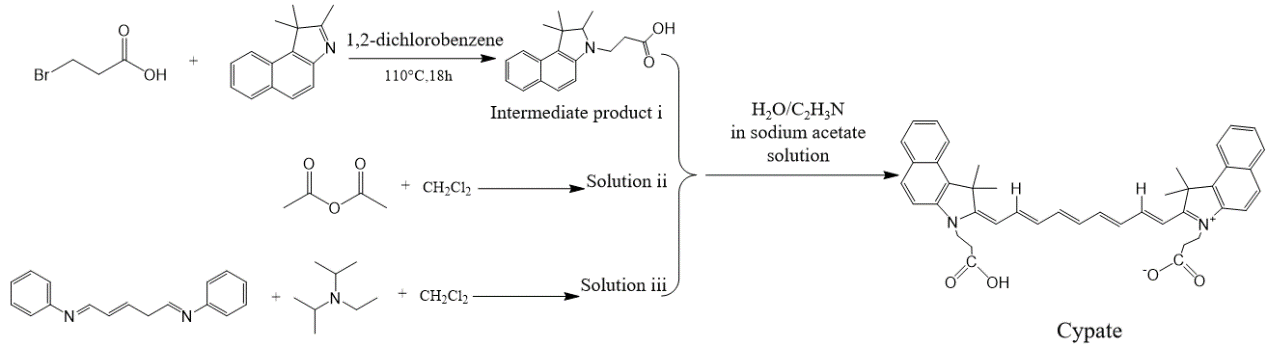


**Scheme S1.** Synthesis route of cypate.


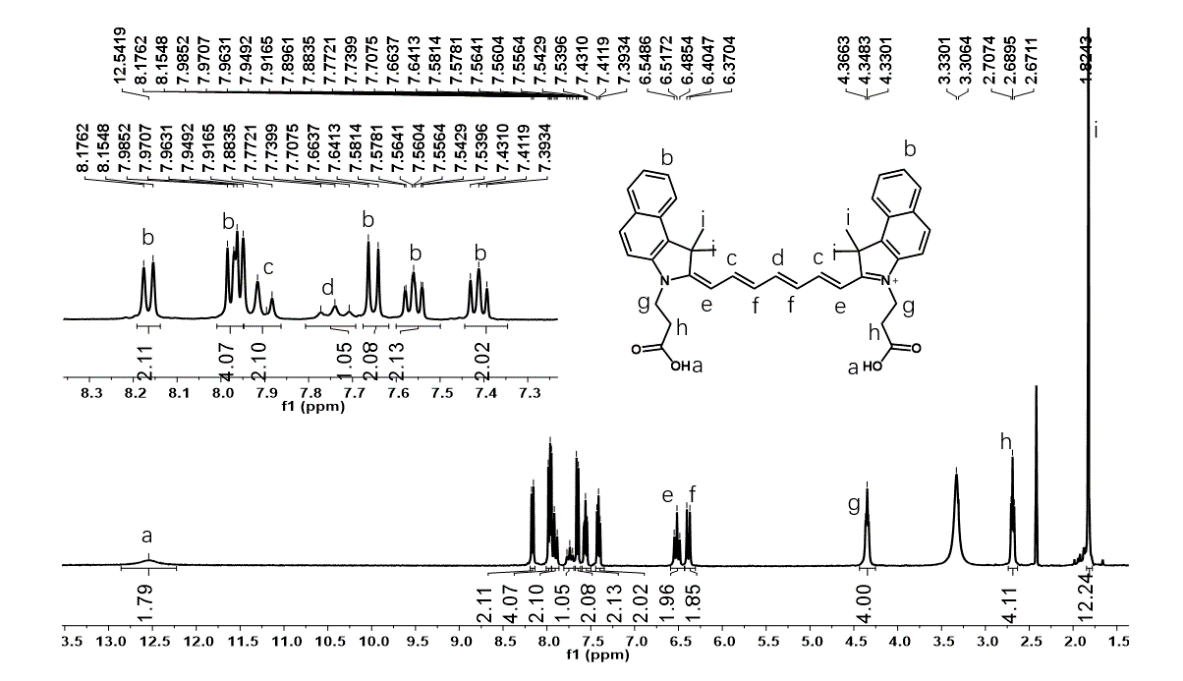


**Figure S1.** H-NMR spectrum of cypate in DMSO-d^6^. ^1^H NMR (400 MHz, DMSO-*d*_6_) δ 12.54 (s, 2H), 8.17 (d, *J* = 8.6 Hz, 2H), 7.97 (dd, *J* = 8.7, 5.7 Hz, 4H), 7.90 (d, *J* = 13.2 Hz, 2H), 7.74 (t, *J* = 12.9 Hz, 1H), 7.65 (d, *J* = 9.0 Hz, 2H), 7.56 (ddd, *J* = 8.3, 6.8, 1.3 Hz, 2H), 7.44 – 7.35 (m, 2H), 6.52 (t, *J* = 12.6 Hz, 2H), 6.39 (d, *J* = 13.7 Hz, 2H), 4.35 (t, *J* = 7.2 Hz, 4H), 2.69 (t, *J* = 7.3 Hz, 4H), 1.82 (s, 12H).


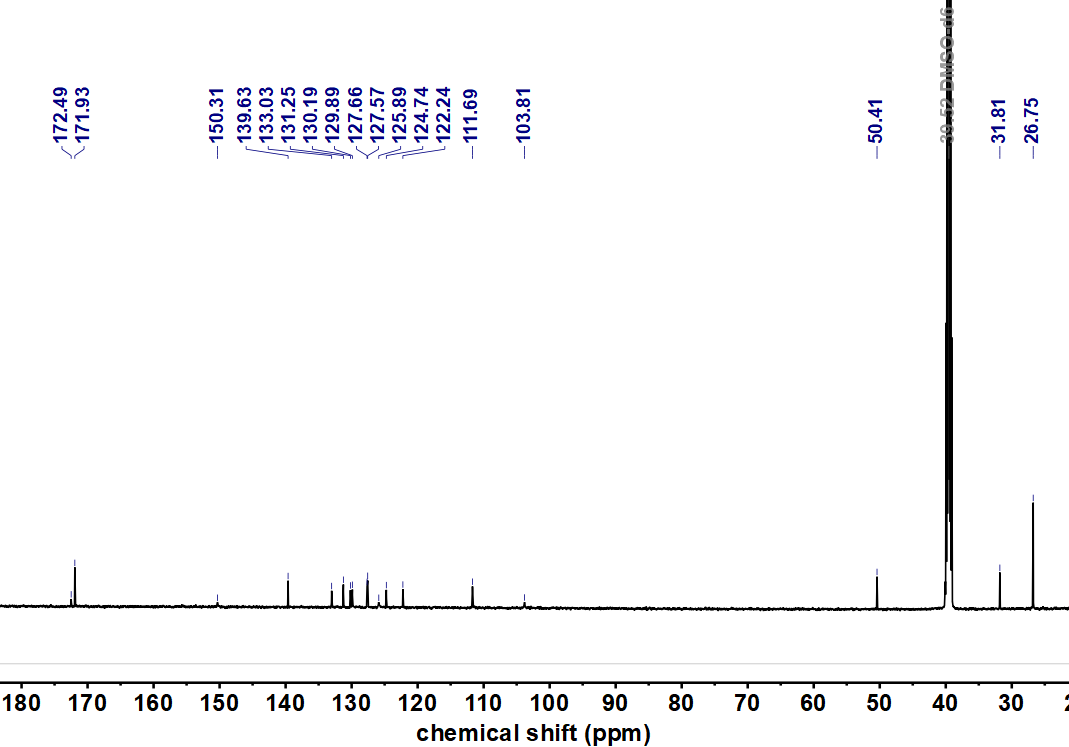


**Figure S2.** ^13^C-NMR spectrum of cypate in DMSO-d^6^.


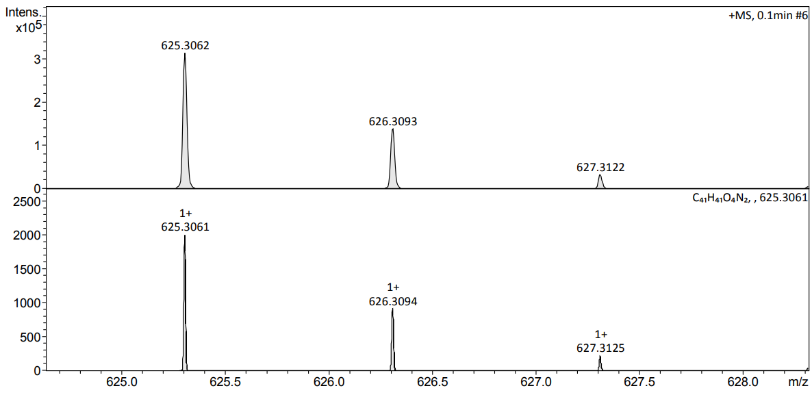


**Figure S3.** Mass spectrometry (MS) of cypate. MS (MALDI-TOF-MS): calcd for C_41_H_41_N_2_O_4_^+^ 625.3061 [M]^+^; found 625.3061 [M]^+^.


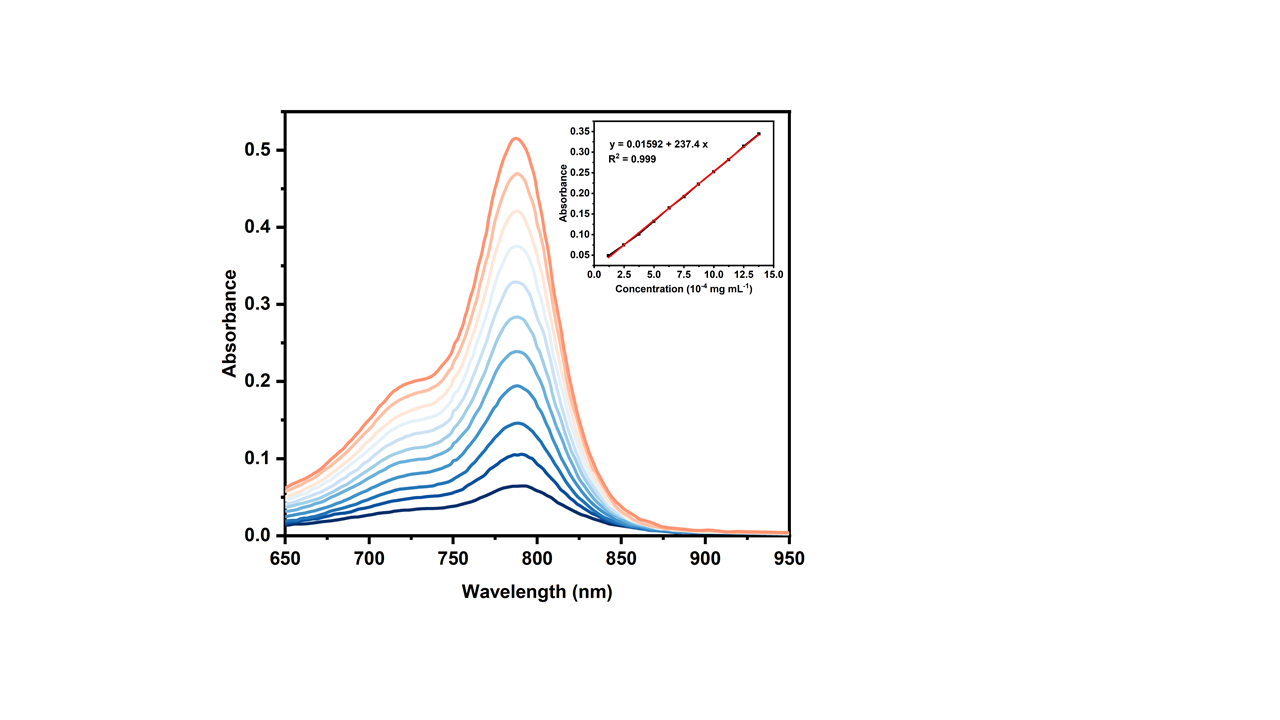


**Figure S4.** Absorption spectra of cypate with different concentrations of 0.0001-0.0015 mg mL^-1^. Inset: absorbance at 808 nm as a function of cypate concentration. The molar extinction coefficient of cypate at 808 nm was calculated to be 165756 M^-1^cm^-1^. The standard curve was used for quantitative determination of unbound cypate in the supernatant after cypate-NPs formation. The exact amount of cypate in cypate-NPs was calculated by subtracting the amount in the supernatant from the initial known amount.


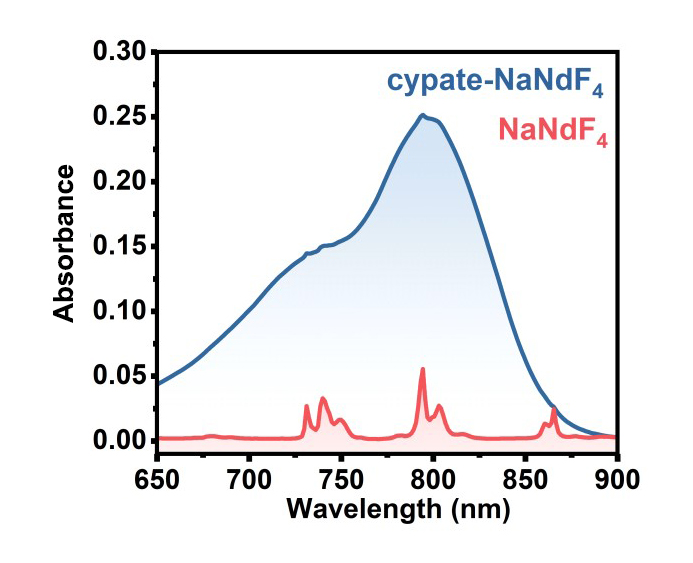


**Figure S5.** Absorbances of NaNdF_4_ and cypate-NaNdF_4_.

**
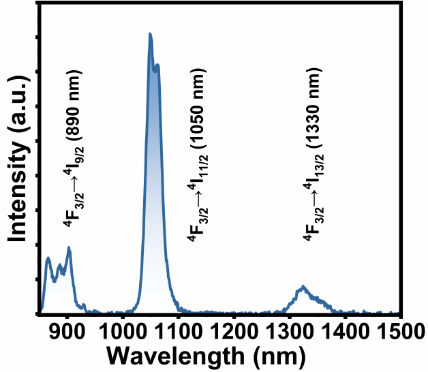
**

**Figure S6.** Emission of a colloidal dispersion of NaNdF_4_ under excitation at 808 nm.


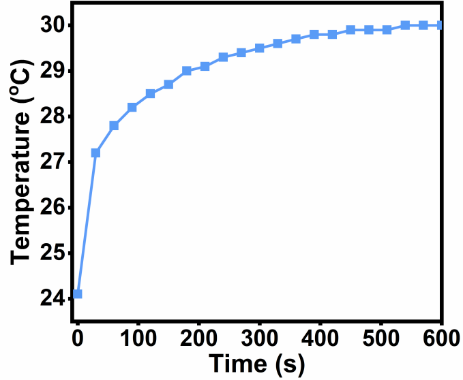


**Figure S7.** Time-dependent temperature curves of NaNdF_4_ dispersion under 808 nm laser irradiation.

**
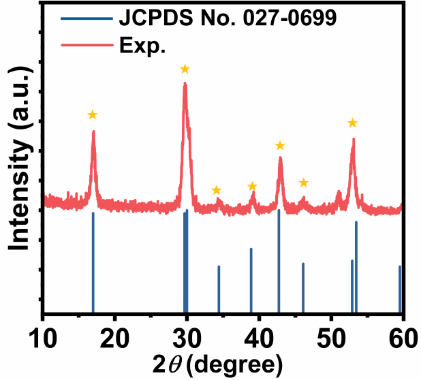
**

**Figure S8.** XRD pattern of NaGdF_4_. All diffraction peaks matched well with the standard pattern of hexagonal *β*-NaGdF_4_ (JCPDS No. 27-0699).

**
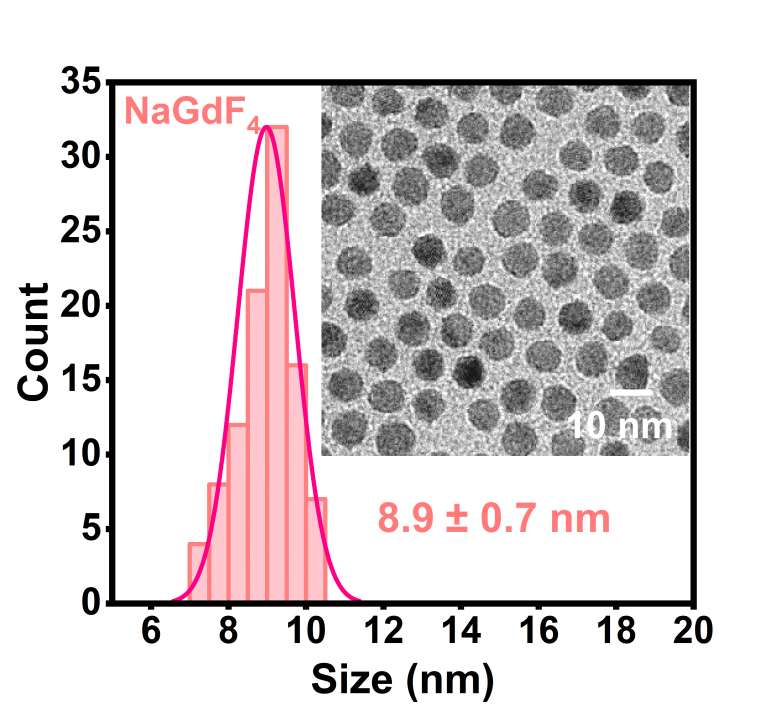
**

**Figure S9.** TEM image of NaNdF_4_ NPs and size distribution histogram by randomly calculating 100 particles in the TEM image.

**
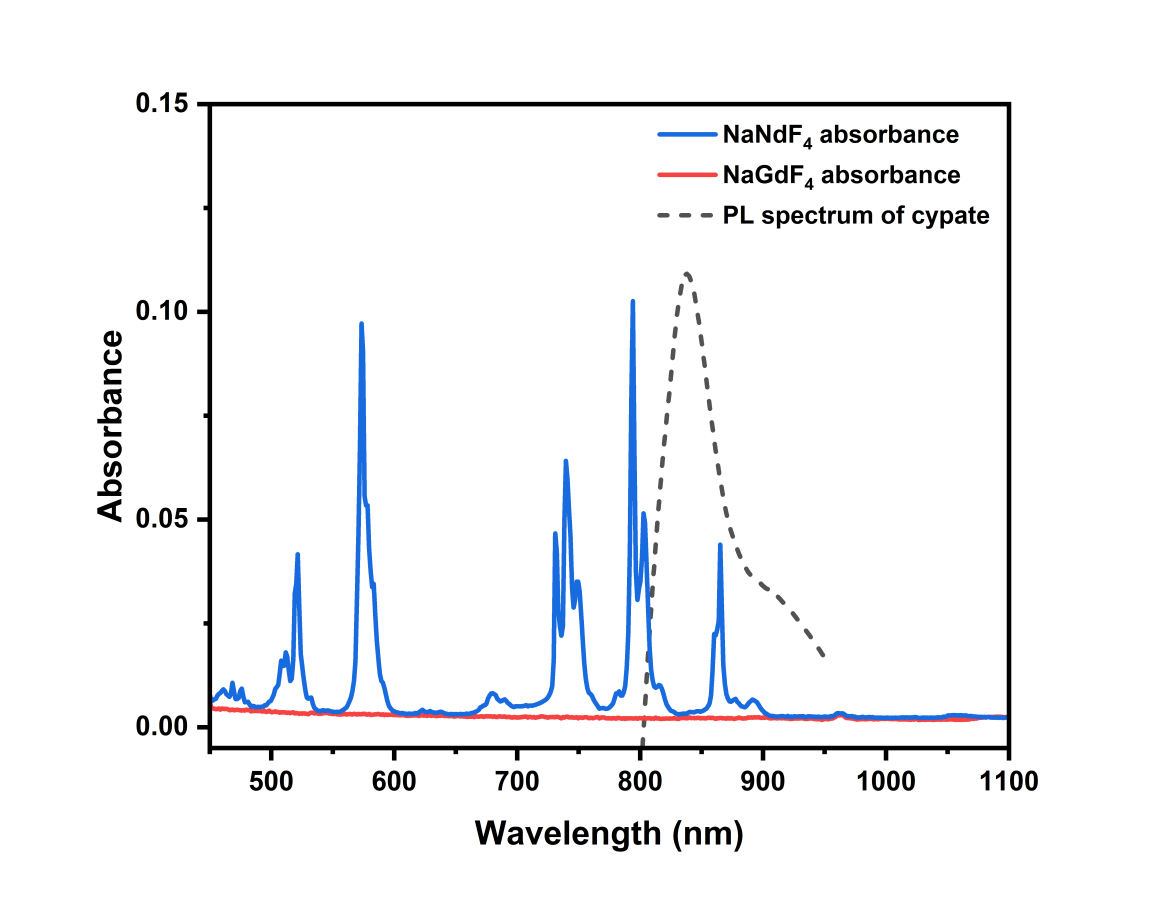
**

**Figure S10.** Absorption spectra of NaNdF_4_ and NaGdF_4_. NaGdF_4_ has no absorption at the emission wavelength of cypate and can serve as an optically inert nanocrystal.


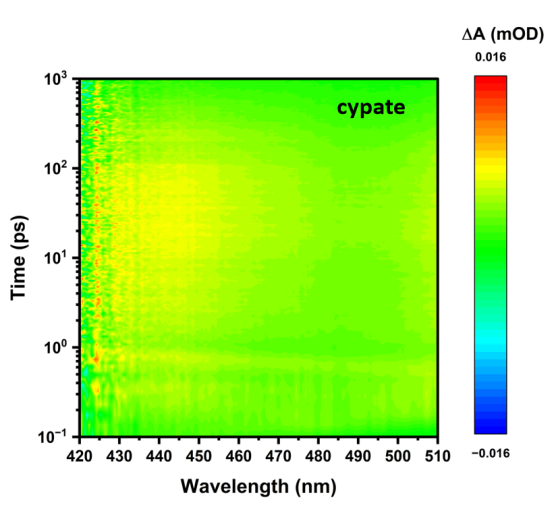


**Figure S11.** fs-TA map with the wavelength ranging from 420-510 nm of cypate pumped at 790 nm.


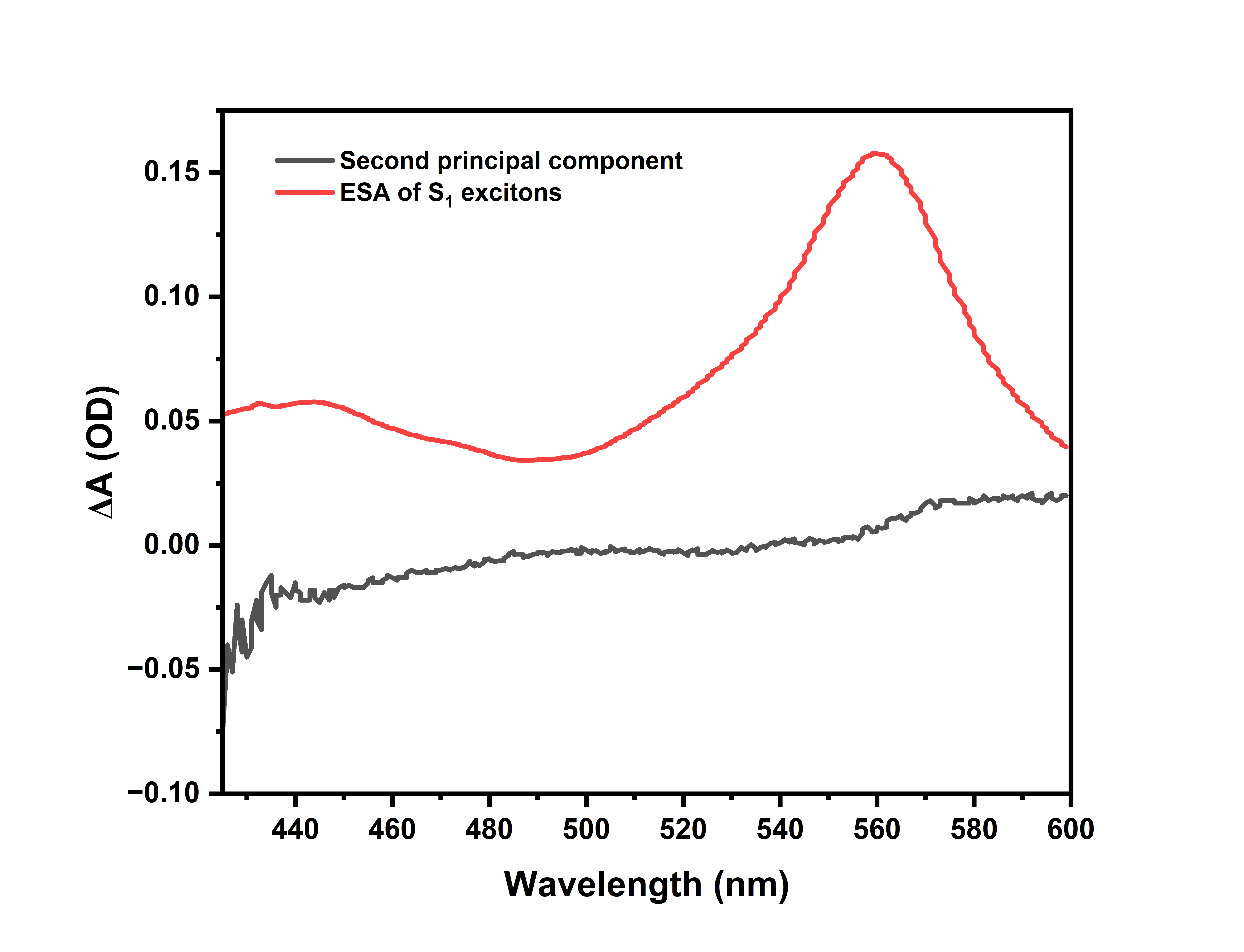


**Figure S12**. Singular value decomposition (SVD) analysis of cypate in the spectral range of 425-600 nm.


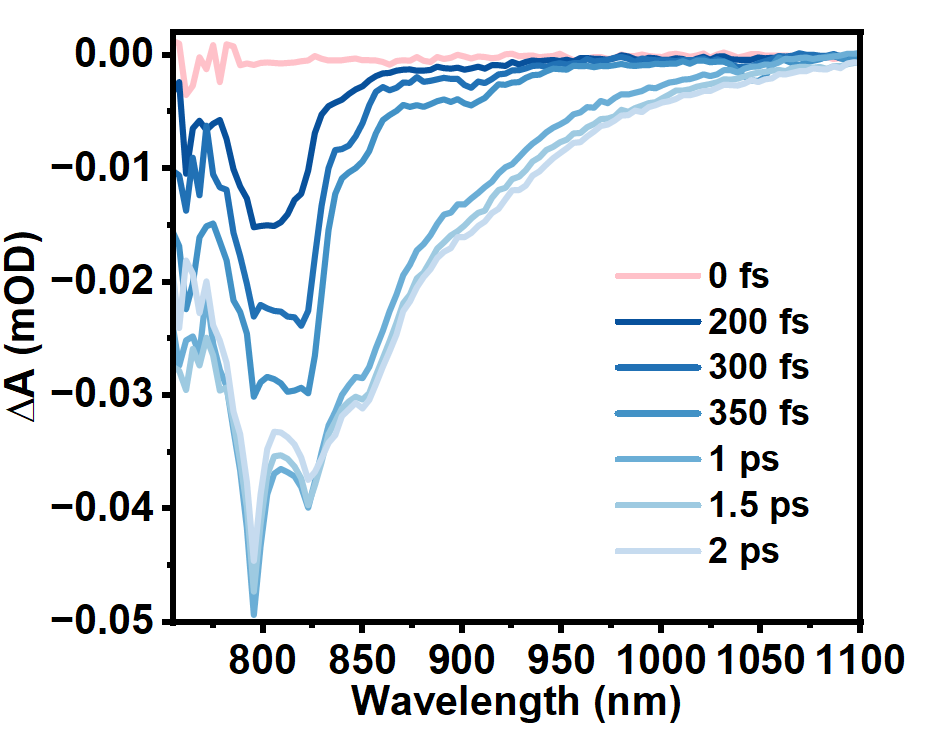


**Figure S13.** Representative time-resolved spectra ranging from 755 to 1100 nm of cypate within the first 2 ps.


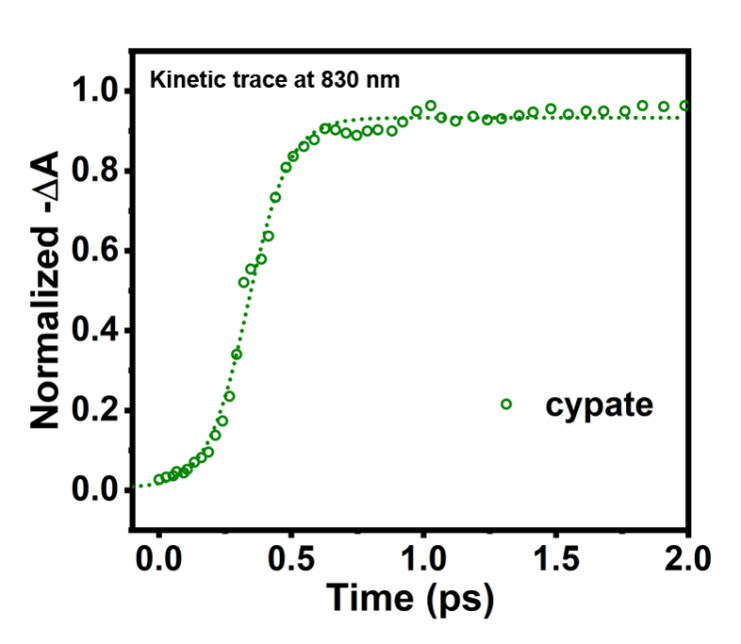


**Figure S14.** TA kinetic trace of cypate probed at 830 nm within the first 2 ps, illustrating the rapid formation of S_1_ excitons.


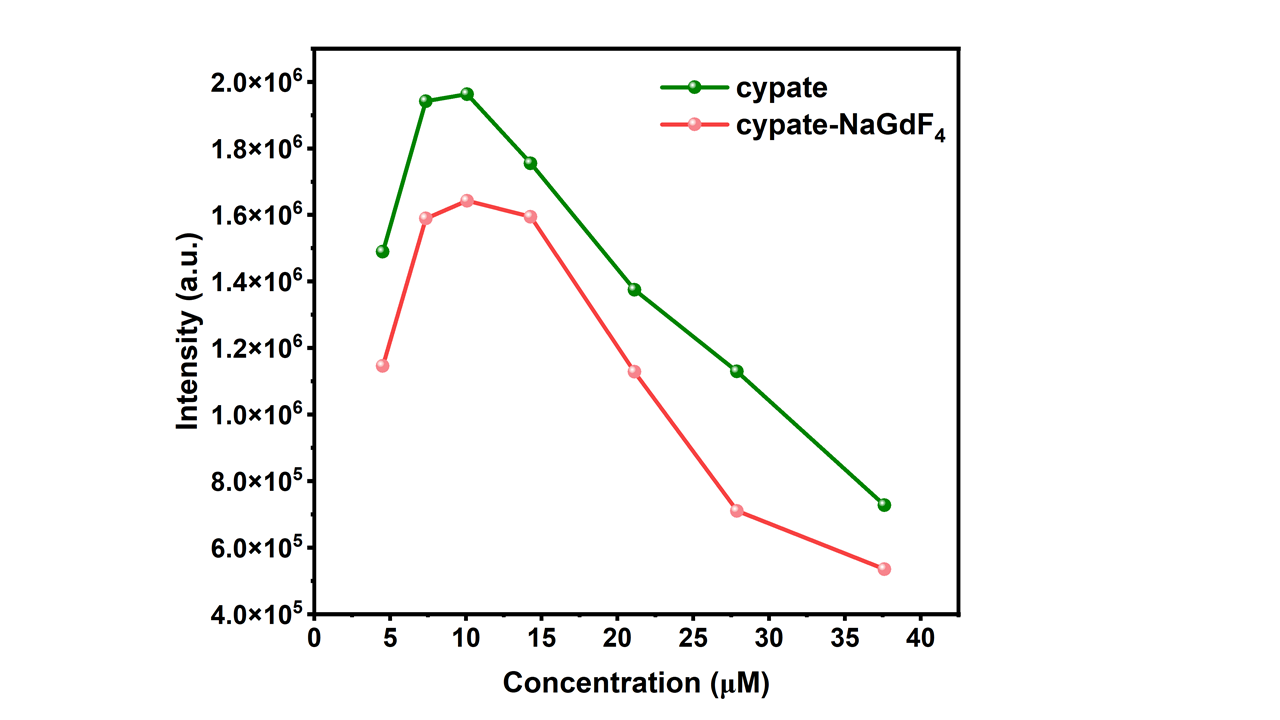


**Figure S15.** Integrated emission intensity (800-950 nm) of cypate and cypate-NaGdF_4_. Due to the enhancement of intermolecular interactions, the binding of cypate on NaGdF_4_ quenched part of fluorescence and promoted heat generation.

**
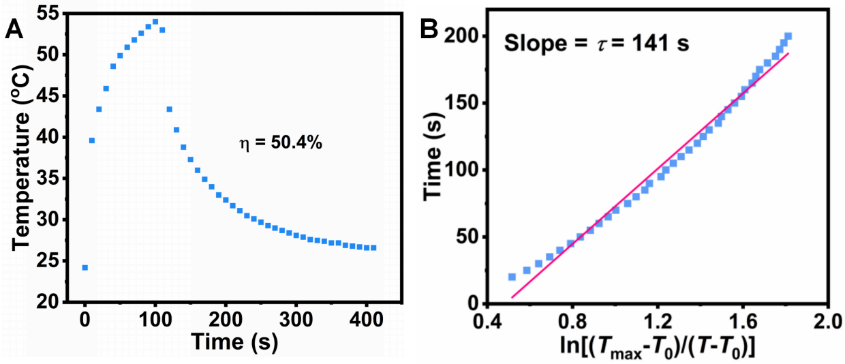
**

**Figure S16.** (A) Temperature profile of cypate-NaNdF_4_ composed of 21 $\mu$M cypate and 1 mg mL^-1^ NaNdF_4_ irradiated with the 808-nm laser. (B) Determination of time constant using linear regression of the cooling profile in (A).


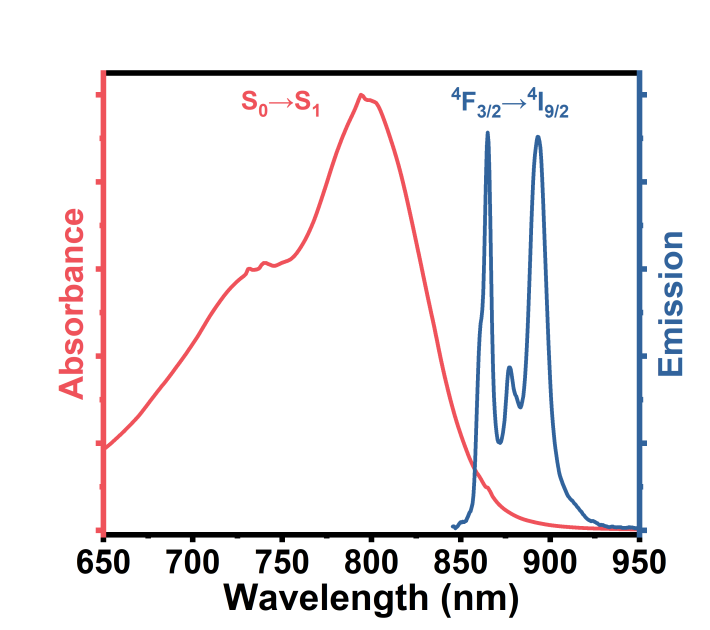


**Figure S17.** Emission spectrum of NaNdF_4_ and absorption spectrum of cypate.


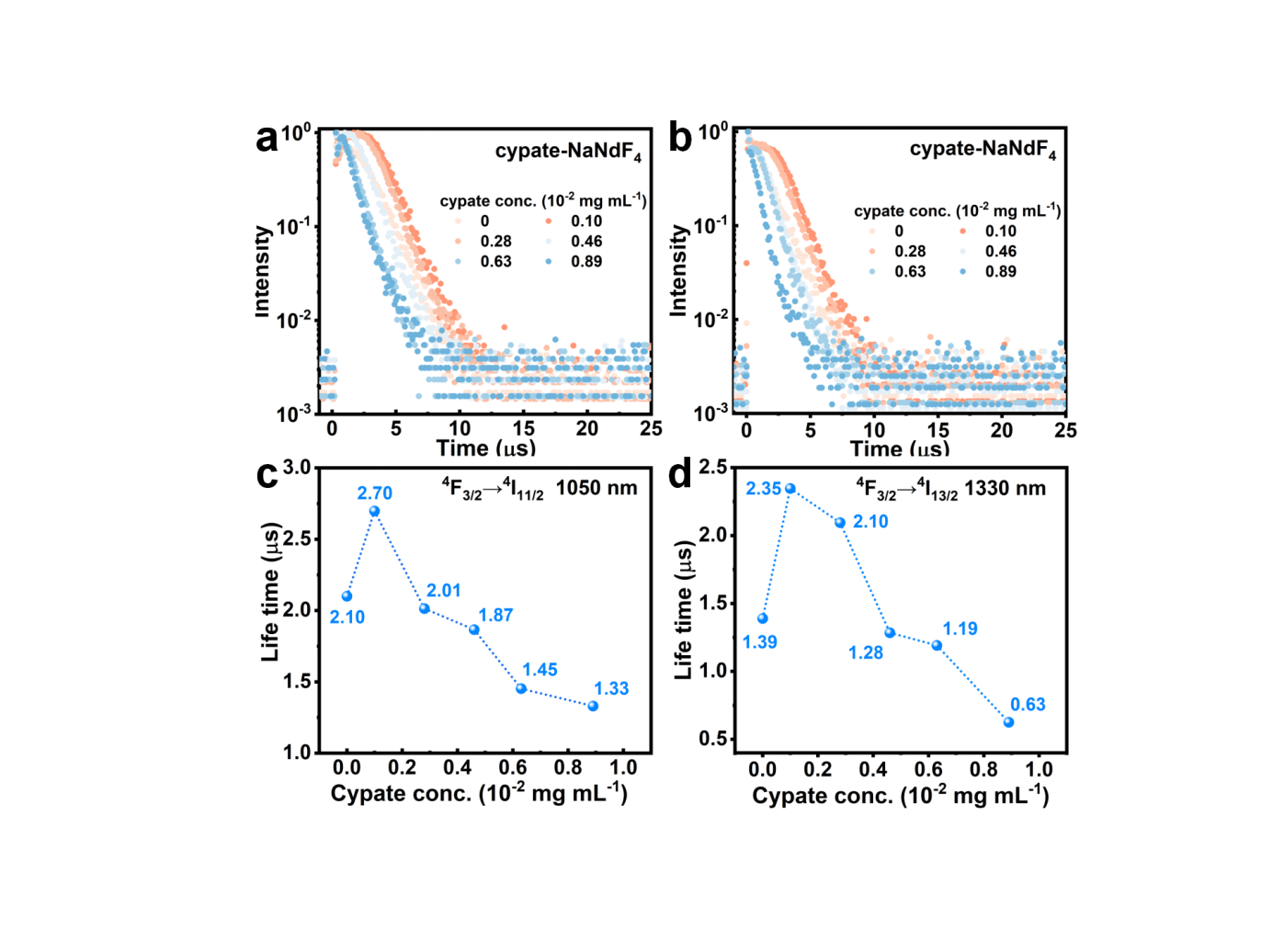


**Figure S18.** PL decay kinetic curves of Nd^3+^ loaded with different amount of cypate at (a) 1050 nm and (b) 1330 nm respectively. PL decay lifetimes at (c) 1050 nm and (d) 1330 nm respectively.


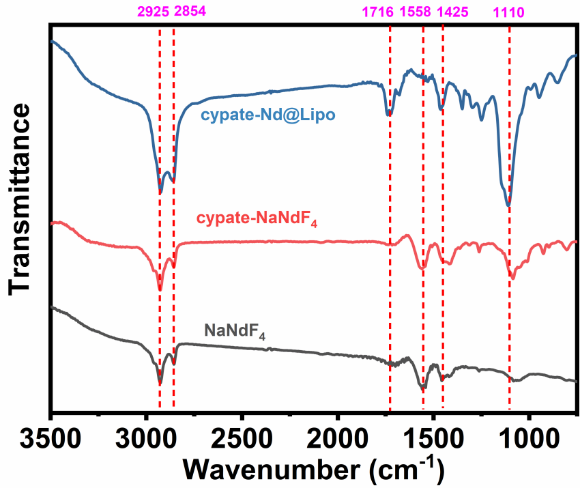


**Figure S19.** FT-IR spectra of NaNdF_4_, cypate-NaNdF_4_, and cypate-Nd@Lipo. The peaks at 2925 and 2854 cm^−1^ were attributed to the asymmetric and symmetric stretching vibrations of methylene (–CH_2_–) in the long alkyl chain. In addition, peaks at 1558 cm^-1^ and 1425 cm^-1^ were associated with the asymmetric and symmetric stretching vibrations of the carboxylic group of the bound oleate acid ligands. After combining with DSPE-PEG, the peaks at 2925 and 2854 cm^−1^ were significantly weakened, and a strong and sharp absorption peak at 1110 cm^−1^ appeared, indicating that cypate-Nd@Lipo was successfully obtained.


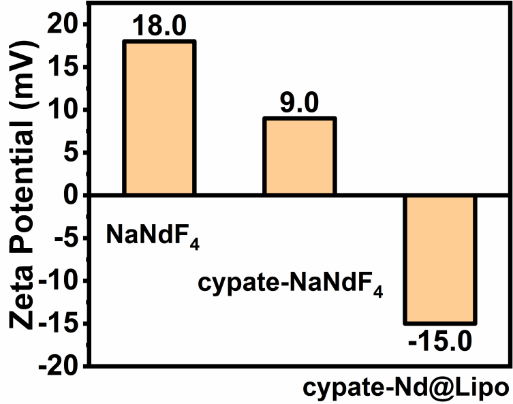


**Figure S20.** Zeta potentials of NaNdF_4_, cypate-NaNdF_4_, and cypate-Nd@Lipo.

**
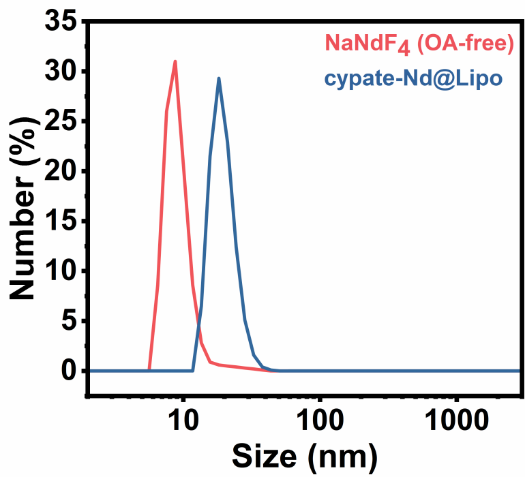
**

**Figure S21.** Particle size distributions of NaNdF_4_ and cypate-Nd@Lipo dispersed in water.

**References**

[1] Z. Yu, W. Hu, H. Zhao, X. Miao, Y. Guan, W. Cai, Z. Zeng, Q. Fan, T. T. Y. Tan, *Angew. Chemie - Int. Ed.* **2019**, *58*, 8536.

[2] W. Zou, C. Visser, J. A. Maduro, M. S. Pshenichnikov, J. C. Hummelen, *Nat. Photonics* **2012**, *6*, 560.

[3] Y. Wang, D. Liu, M. You, H. Yang, H. Ke, *J. Mater. Chem. B* **2022**, *10*, 3016.

[4] M. Zheng, P. Zhao, Z. Luo, P. Gong, C. Zheng, P. Zhang, C. Yue, D. Gao, Y. Ma, L. Cai, *ACS Appl. Mater. Interfaces* **2014**, *6*, 6709.

[5] L. Zhang, X. Q. Yang, J. S. Wei, X. Li, H. Wang, Y. Di Zhao, *Theranostics* **2019**, *9*, 5424.

[6] P. Huang, J. Lin, W. Li, P. Rong, Z. Wang, S. Wang, X. Wang, X. Sun, M. Aronova, G. Niu, R. D. Leapman, Z. Nie, X. Chen, *Angew. Chemie - Int. Ed.* **2013**, *52*, 13958.

[7] J. Zeng, D. Goldfeld, Y. Xia, *Angew. Chemie* **2013**, *125*, 4263.

[8] C. M. Hessel, V. P. Pattani, M. Rasch, M. G. Panthani, B. Koo, J. W. Tunnell, B. A. Korgel, *Nano Lett.* **2011**, *11*, 2560.

[9] K. C. Li, H. C. Chu, Y. Lin, H. Y. Tuan, Y. C. Hu, *ACS Appl. Mater. Interfaces* **2016**, *8*, 12082.

[10] T. Yang, Y. Tang, L. Liu, X. Lv, Q. Wang, H. Ke, Y. Deng, H. Yang, X. Yang, G. Liu, Y. Zhao, H. Chen, *ACS Nano* **2017**, *11*, 1848.

[11] J. Liu, X. Zheng, L. Yan, L. Zhou, G. Tian, W. Yin, L. Wang, Y. Liu, Z. Hu, Z. Gu, C. Chen, Y. Zhao, *ACS Nano* **2015**, *9*, 696.

[12] H. Lin, S. Gao, C. Dai, Y. Chen, J. Shi, *J. Am. Chem. Soc.* **2017**, *139*, 16235.

[13] H. Lin, Y. Wang, S. Gao, Y. Chen, J. Shi, *Adv. Mater.* **2018**, *30*, 1.

[14] H. Lin, X. Wang, L. Yu, Y. Chen, J. Shi, *Nano Lett.* **2017**, *17*, 384.

[15] W. He, K. Ai, C. Jiang, Y. Li, X. Song, L. Lu, *Biomaterials* **2017**, *132*, 37.

[16] X. Cai, X. Jia, W. Gao, K. Zhang, M. Ma, S. Wang, Y. Zheng, J. Shi, H. Chen, *Adv. Funct. Mater.* **2015**, *25*, 2520.

[17] M. Qiu, D. Wang, W. Liang, L. Liu, Y. Zhang, X. Chen, D. K. Sang, C. Xing, Z. Li, B. Dong, F. Xing, D. Fan, S. Bao, H. Zhang, Y. Cao, *Proc. Natl. Acad. Sci. U. S. A.* **2018**, *115*, 501.

[18] M. Zhang, F. Wu, W. Wang, J. Shen, N. Zhou, C. Wu, *Chem. Mater.* **2019**, *31*, 1847.

[19] P. Zhang, H. Huang, J. Huang, H. Chen, J. Wang, K. Qiu, D. Zhao, L. Ji, H. Chao, *ACS Appl. Mater. Interfaces* **2015**, *7*, 23278.

[20] Q. Tian, Y. Li, S. Jiang, L. An, J. Lin, H. Wu, P. Huang, S. Yang, *Small* **2019**, *15*, 1.
